# Steric Selection of Anion Binding Sites by Organoantimony(V) Pnictogen Bond Donors: An Experimental and Computational Study

**DOI:** 10.1021/acs.inorgchem.4c03178

**Published:** 2024-12-03

**Authors:** Brendan L. Murphy, Logan T. Maltz, François P. Gabbaï

**Affiliations:** Department of Chemistry, Texas A&M University, College Station, Texas 77843-3255, United States

## Abstract

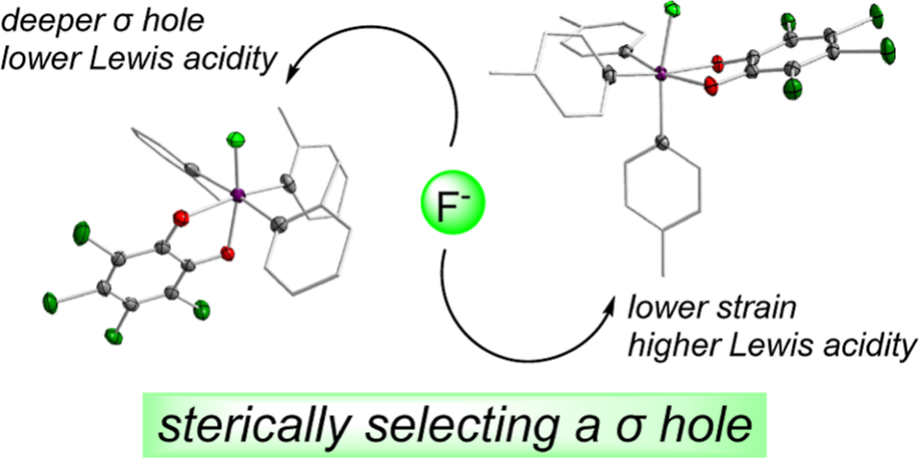

Catecholatostiboranes have emerged as useful Lewis acids in several applications. To better understand the factors that control the properties of these species, we examined the Lewis acidities of (*o*-C_6_Cl_4_O_2_)Sb(*o*-Tol)_3_ (**2**, Tol = tolyl) and (*o*-C_6_Cl_4_O_2_)Sb(*p*-Tol)_3_ (**3**), two triarylcatecholatostiboranes that differ by the nature of the aryl substituent. Fluoride anion binding studies indicate that **3** is more Lewis acidic than **2**, a factor readily assigned to the steric crowding around antimony in the case of the *o*-tolyl derivative. But, while **3** binds F^–^ trans to a Sb–C_aryl_ bond as is typical of catecholatostiboranes, **2** prefers binding trans to a Sb–O bond. Computational analyses of **2** and **3** reveal the existence of several σ holes, and an activation strain analysis is employed to elucidate the origin of these stiboranes’ anion-binding geometry preferences.

## Abstract 2

Two neutral antimony(V) Lewis acids have been synthesized and their fluoride binding behaviors have been thoroughly examined. Their fluoride adducts display quite different geometries with respect to the location of the bound anion. The origin of this difference has been explored computationally.

## Introduction

Central to the fundamental chemistry of Lewis acids is their ability to engage a Lewis base (LB) *via* a dative interaction. Group 13 compounds, particularly those containing boron which engage LBs with an empty *p*-orbital,^1^ are considered prototypical Lewis acids and have found numerous uses in fields like bond activation chemistry.^2,3^ That said, a large body of research has shown that many heavy *p*-block elements also possess inherently high Lewis acidic properties,^4−7^ which have recently found use for anion recognition,^8^ anion transport^9^ as well as catalysis.^10^ Among these main group centers, antimony^11^ stands out as providing particularly strong pnictogen-centered Lewis acidic interactions, or “pnictogen bonds” (PnBs).^12^ Such PnBs are facilitated by LBs interacting with low-lying empty σ* orbitals and their accompanying regions of high electropositive character (σ holes), as well as flexible bonds that help reduce the steric pressure of LB binding.^13^

The strength of antimony-centered PnBs can be augmented on several fronts,^14−17^ but is most easily achieved by the oxidation of the antimony center from the +III to the +V state.^16^ In our group, we have found it convenient to access the +V state by reacting stibines with a highly oxidizing *ortho*-quinone like *o*-chloranil to yield catecholatostiboranes.^15,18−20^ In general, these catecholatostiboranes are water-tolerant^21,22^ yet are sufficiently Lewis acidic to engage with basic substrates^21−27^ and anions like fluoride^15,19,20^ and hydroxide.^15,28^ Since then, these catecholatostiboranes have proven to be particularly useful for the study and application of highly Lewis acidic platforms with enhanced Lewis acidic properties compared to their trivalent counterparts.^16,17,20,27,28^

**Figure 1:**
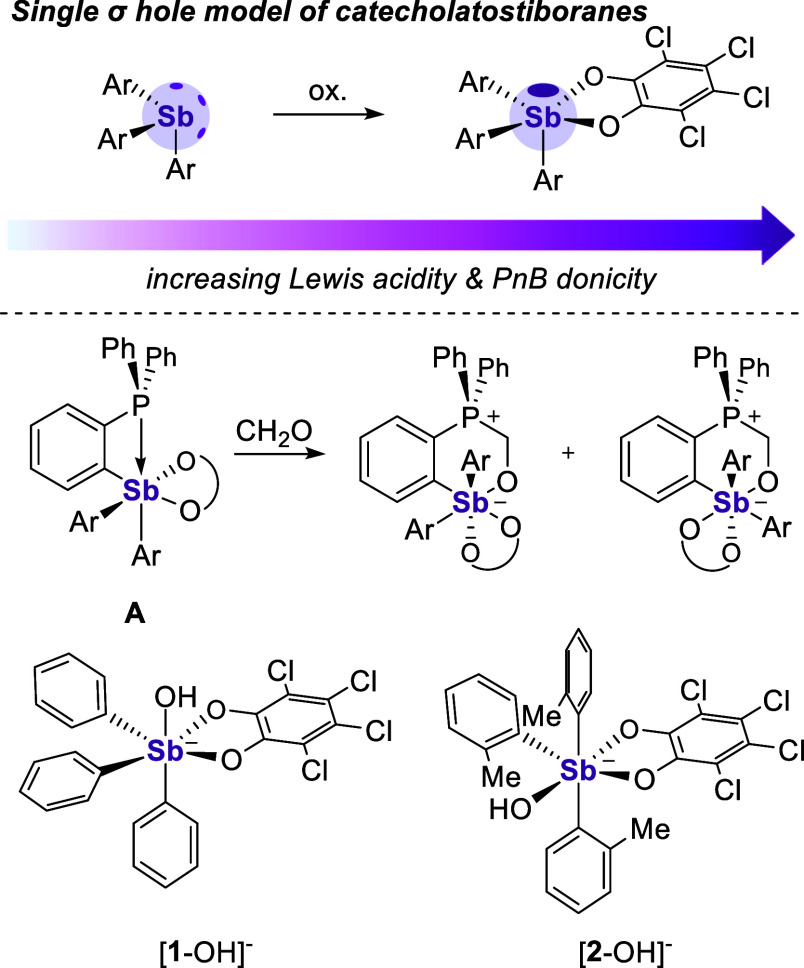
Top: visualization of the single σ hole model of the Lewis acidity of catecholatostiboranes. Bottom: catecholatostiboranes that challenge the single σ hole model. The tetrachlorophenylene backbone of the catecholate ligands in **A** is represented by a curved line.

One model posited to explain the enhancement of PnB donor properties of catecholatostiboranes arises from the deepening of σ holes on the antimony surface in the +V state compared to the +III state.^16^ Coincidentally, crystallographic studies suggest a predilection of catecholatostiboranes for pseudo-square pyramidal (SP, τ_5_^29^ < 0.5) geometries^19^ with a Sb–C_aryl_ bond defining the apex when accepting LBs. Models emphasizing the σ hole typically combine these two observations, and explain the apparent existence of a single static Lewis acidic site on a catecholatostiborane as arising from the coalescence of three shallower σ holes on the triarylstibine surface upon oxidation (Figure 1).^16,17,22^

While this “single σ hole” model is simple and may sufficiently predict the Lewis acidic behavior of most catecholatostiboranes, we have recently noticed some inconsistencies with it. Most fundamentally, given that there are five primary bonds to the antimony center, the potential for multiple σ holes exists in these derivatives. Moreover, it is surprising that in a model that emphasizes the electrostatics of a PnB, the only σ hole is found trans to a Sb–C_aryl_ bond and not trans to a more polar Sb–O_*o*-chloranil_ bond.^14,31−33^ Additionally, we find that free catecholatostiboranes are quite flexible^13^ and tend to take on pseudo-trigonal bipyramidal geometries (TBP, τ_5_ > 0.5) in the solid-state, which reveals the binding site found trans to a Sb–O bond.^17,28,34^ Indeed, there are several instances in which the LB-accepting geometry proposed by the single σ hole model (i.e., LB → Sb–C_aryl_) is not the thermodynamically favored conformation. For instance, we have spectroscopically and crystallographically observed the O → Sb complexation and activation of a formaldehyde molecule trans to an Sb–O_*o*-chloranil_ bond of stiborane **A**, suggesting the existence of multiple regions of PnB donicity (Figure 1).^21^ This is supported by our recent study of hydroxoantimonates in which we find that **1** binds the nucleophilic hydroxide anion in accordance with single σ hole model while its more sterically encumbered sibling **2** does not (Figure 1).^28^

As these catecholatostiboranes find utility beyond LB complexation chemistry, it is important to interrogate working models like the single σ hole model in order to explain these compounds’ Lewis acidities more accurately. Thus, we have decided to thoroughly examine two related catecholatostiboranes experimentally and computationally. Our findings reiterate that *ortho*-positioned methyl groups on the aryl rings direct anion binding, in this case the binding of fluoride, to a site on the antimony surface not prescribed by the single σ hole model. This *ortho*-substitution also dampens the PnB donicity of the catecholatostiborane center compared to its *para*-substituted sibling, though both compounds are still capable of binding fluoride in competing media. We then buttress these findings with computational analysis that reveals the presence of several σ holes on the antimony surface. Following this, we perform energy decomposition analysis calculations on the fluoroantimonate complexes and find that their geometric binding preferences are not guided by the single σ hole model. Instead, their binding preferences arise from a delicate trade-off between energy associated with the free receptor straining to accept the fluoride anion at a particular site and the strength of the F^–^ → Sb interaction at different σ holes.

## Results and Discussion

### Synthesis and Characterization

**Figure 2:**
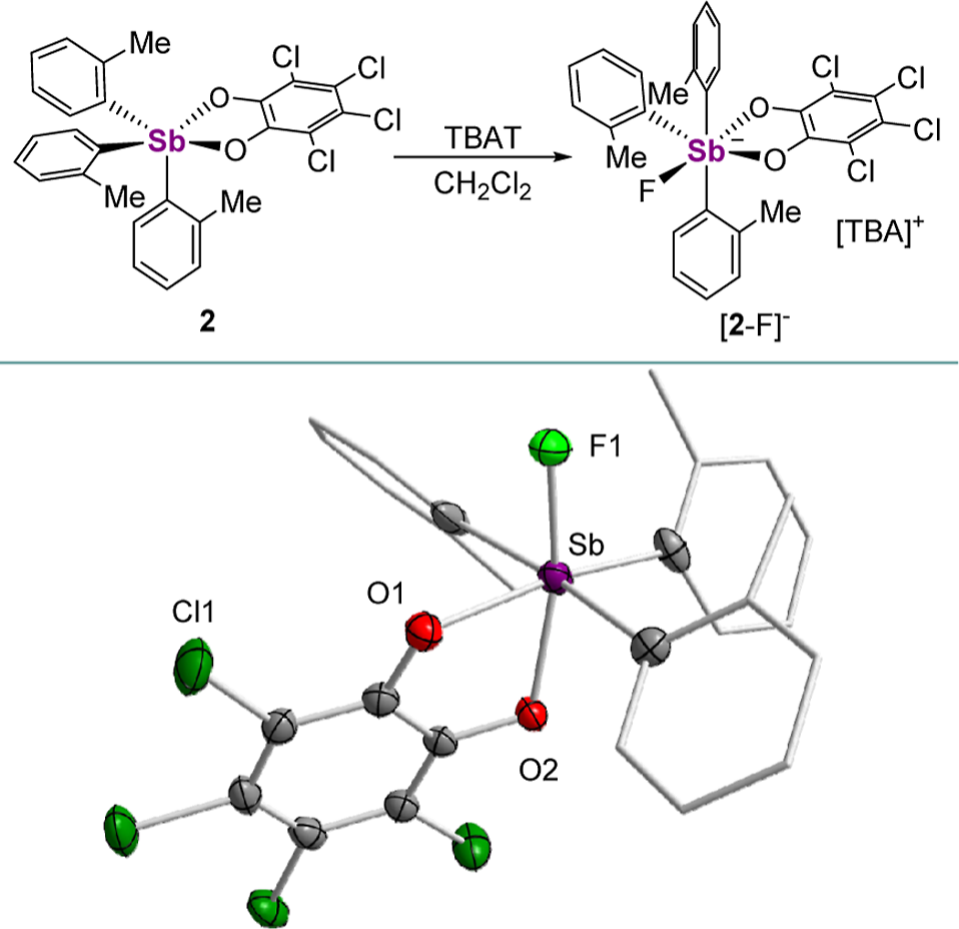
Top: synthesis of [^*n*^Bu_4_N][**2**-F]. Bottom: solid-state structure of [**2**-F]^−^. Disorder at two of the *o*-Tol rings, hydrogen atoms, and [^*n*^Bu_4_N]^+^ counterion are omitted for the sake of clarity. Selected bond lengths (Å) and angles (deg) for [**2**-F]^−^: Sb–F_1_: 1.9758(15), Sb–O_1_: 2.1621(16), Sb–O_2_: 2.08013(16), F_1_–Sb–O_2_: 165.12(7), O_1_–Sb–O_2_: 77.59(16).

To begin, the previously reported compound **2**(28) was reacted with one equivalent of [^*n*^Bu_4_N][Ph_3_SiF_2_] (TBAT) in CH_2_Cl_2_. Crystallization *via* layering with hexanes afforded [^*n*^Bu_4_N][**2**-F] as confirmed by X-ray and elemental analysis. The ^1^H NMR spectrum of [^*n*^Bu_4_N][**2**-F] yields a complex spectrum that cannot be assigned based on the formation of a single conformer (Figure S1). This view is supported by the ^19^F NMR spectrum of [^*n*^Bu_4_N][**2**-F] in CDCl_3_, which reveals the presence of three resonances, a sharp peak at −69.17 ppm and two broad resonances at −108.15 and −114.57 ppm. These spectroscopic features were observed on multiple samples, including purified ones, for which a correct elemental analysis was obtained. The structure of [^*n*^Bu_4_N][**2**-F] locates the bound fluoride to be trans to the Sb–O_*o*-chloranil_ bond (Figure 2), a configuration that is nearly identical to that of the hydroxide-bound complex [**2**-OH]^−^.^28^ At 1.9758(15) Å, this Sb–F bond length is similar to that found for [**1**-F]^−^ (1.9877(13) Å).^15^ There is also a notable elongation of the Sb_1_–O_1_ bond compared to the Sb_1_–O_2_ bond (2.1621(16) Å vs 2.0813(16) Å), a phenomenon that we assign to the differing trans-influences of the ligands opposite to the oxygen atoms.

**Figure 3:**
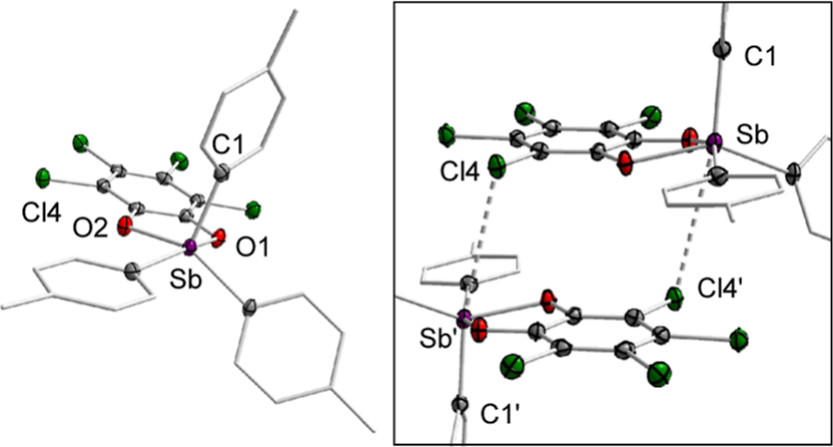
Solid-state structure of **3**. Hydrogen atoms are omitted for the sake of clarity. Inset shows the intermolecular PnBs present in the solid-state. Selected bond lengths (Å) and angles (deg) for **3**: Sb_1_–C_1_ 2.100(2), Sb_1_–O_1_ 2.0980(17), Sb_1_–O_2_ 2.0417(19), Sb–Cl_4_’ 3.7320(6), O_1_–Sb_1_–O_2_ 78.02(7), C_1_–Sb–Cl_4_’ 169.57(6).

For comparison, we also generated stiborane **3**, which is adorned with *p*-Tol groups, by treating the stibine precursor with one equivalent of *o*-chloranil. This bright yellow compound has been fully characterized by multinuclear NMR spectroscopy, X-ray diffraction, and elemental analysis. Compound **3** displays only two aryl resonances in its ^1^H NMR spectrum in CDCl_3_, indicating a fluxional geometry when in solution like **2**. Its solid-state structure reveals a τ_5_ value of 0.27 and is best described as attaining a pseudo-SP geometry. Curiously, this geometry seems to be reinforced by dimeric intermolecular Cl_*o*-chloranil_ → Sb interactions (Figure 3) that are below the sum of the van der Waals radii of the two elements (Σ_vdW_(Sb,Cl) = 4.29 Å).^35^

**Figure 4:**
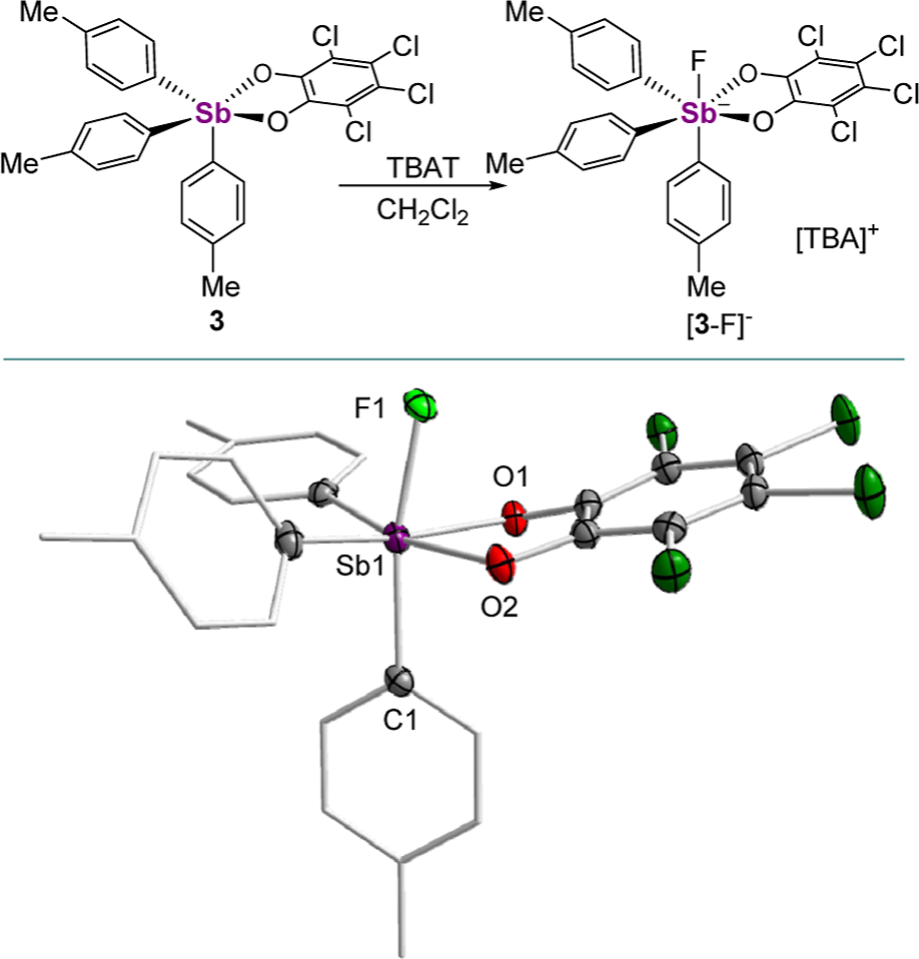
Solid-state structure of [**3**-F]^−^. Hydrogen atoms and [^*n*^Bu_4_N]^+^ counterion are omitted for the sake of clarity. Only one of the two independent molecules found in the cell is shown. Selected bond lengths (Å) and angles (deg) for [**3**-F]^−^: Independent molecule 1. Sb_1_–F_1_ 1.9986(12), Sb_1_–O_1_ 2.0871(14), Sb_1_–O_2_ 2.1024(14), F_1_–Sb_1_–C_1_ 168.95(6), O_1_–Sb_1_–O_2_ 78.21(6). Independent molecule 2. Sb_2_–F_2_ 2.0158(12), Sb_2_–O_3_ 2.0961(14), Sb_2_–O_4_ 2.0975(14), F_2_–Sb_2_–C_44_ 169.24(7), O_3_–Sb_2_–O_4_ 78.43(6).

Treatment of **3** with TBAT in CH_2_Cl_2_ gives rise to [^*n*^Bu_4_N][**3**-F], which by ^1^H NMR spectroscopy display two sets of protons at a 2:1 ratio, suggesting formation of an octahedral fluoroantimonate complex. A resonance at −83.63 ppm in the ^19^F NMR spectrum is also in the range of previously reported fluoroantimonate ions.^15,19^ This notion is confirmed *via* the elucidation of the solid-state structure of [**3**-F]^−^ (Figure 4). Two independent [^*n*^Bu_4_N][**3**-F] molecules are found in the asymmetric unit, though both display slightly longer Sb–F distances (Sb_1_–F_1_: 1.9986(12) Å and Sb_2_–F_2_: 2.0158(12) Å) than either [**1**-F]^−^ or [**2**-F]^−^. That said, both structures find fluoride trans to a Sb–C_*p*-Tol_ bond in accordance with the single σ hole model.

### Fluoride Binding Measurements

**Figure 5:**
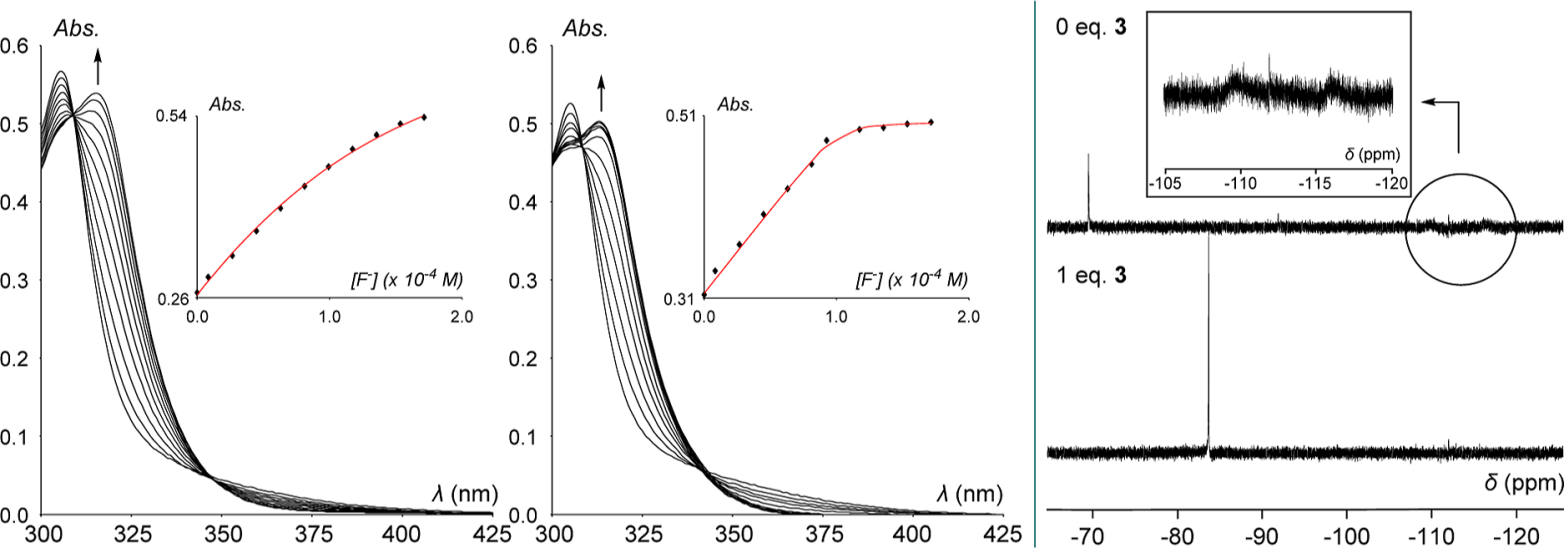
Left: spectral changes in the UV–vis absorption spectra of **2** (1.06 × 10^–4^ M, left) and 3 (1.02 × 10^–4^ M, right) in CHCl_3_ upon incremental addition of a solution of TBAF·3 H_2_O (1.85 × 10^–2^ M) in CHCl_3_. In each case, the associated insets show the experimental (black diamonds) and calculated (red line) 1:1 fluoride binding isotherms for data collected at 316 and 313 nm for **2** and **3**, respectively. These fittings yielded *K*(F^–^) values of 13,000 (±400) M^–1^ (ε(2) = 2500 M^–1^ and ε([**2**-F]^−^) = 6950 M^–1^) for **2** and >10^6^ M^–1^ (ε(3) = 3095 M^–1^ and ε([**3**-F]^−^) = 4950 M^–1^) for **3**. Right: quantitative fluoride transfer from [**2**-F]^−^ to **3** in CDCl_3_ as monitored by ^19^F NMR spectroscopy.

With these compounds in hand, we then set about determining their Lewis acidities. Issues of solubility precluded fluoride titration analysis in aqueous solvent systems like 7:3 THF:water (v/v), and thus we conducted fluoride titrations with TBAF·3 H_2_O in CHCl_3_. As seen in Figure 5, fitting the resulting data to 1:1 binding isotherms provided their fluoride association constants (*K*(F^–^)). Beginning with **2**, whereas neutral triarylborane species typically show no affinity for fluoride in CHCl_3_,^36^**2** shows a modest affinity for the fluoride anion in this medium with a *K*(F^–^) value of 13,000 (±400) M^–1^. In stark contrast, **3** displays quantitative binding (*K*(F^–^) > 10^6^ M^–1^). Moreover, when [**2**-F]^−^ was challenged with one equivalent of **3** in CDCl_3_, quantitative fluoride transfer to **3** was observed by ^19^F NMR spectroscopy (Figure 5) while [**3**-F]^−^ persisted when challenged with one equivalent of **2** (Figure S8). Thus, we confidently conclude that while both compounds have a strong affinity for the fluoride anion, **3** is more Lewis acidic than **2** by at least two orders of magnitude.^28^

### Computational Results

**Figure 6:**
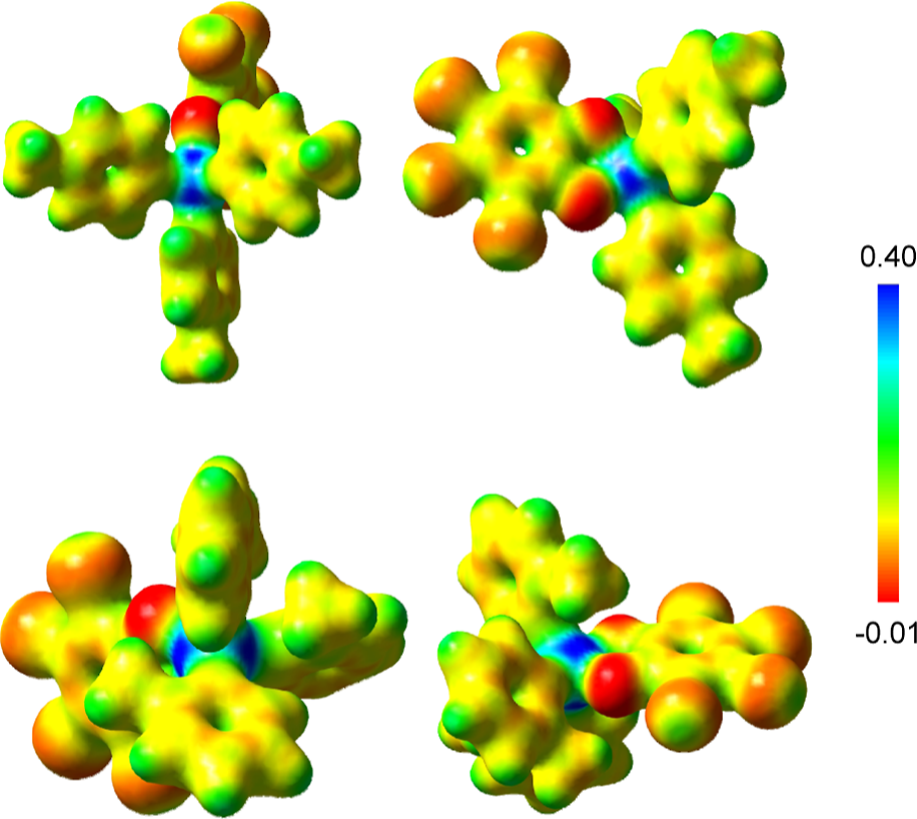
Top: front and side views of the ESP map of **3** (isovalue: 0.03 a.u., gradient scale values given in a.u.) Bottom: front and side views of the ESP of **2** (isovalue: 0.03 a.u., gradient scale values given in a.u.).

To further interrogate these compounds’ Lewis acidic properties, we performed computational analyses, starting with the optimization of the solid-state geometries of **2**, **3**, and their respective fluoride adducts *via* density functional theory methods. Upon optimizing **2** and **3**, we noticed a considerable relaxation from pseudo-SP geometries from the solid-state to more pseudo-TBP geometries (τ_5,calcd_ = 0.46 and 0.76, respectively). Visualization of the electrostatic potential (ESP) map of the less encumbered **3**, which provides the location of its σ holes, showed no regions of electropositive character on the antimony surface when drawn at an isovalue of 0.001. But at a greater isovalue of 0.03, the ESP map of **3** revealed the presence of several σ holes: three found trans to Sb–C_*p*-Tol_ primary bonds and one found trans to a Sb–O_*o*-chloranil_ bond (Figure 6). This finding mirrors that of Naka and co-workers in their study of the Lewis acidity of (o-C_6_Cl_4_O_2_)AsPh_3_ which also identified multiple distinct σ holes on the As(V) surface.^34^ A similar ESP analysis of **2** at this isovalue (0.03) also identifies regions of electropositive character, but these regions are covered by the methyl groups of the *o*-Tol ligands.

**Figure 7:**
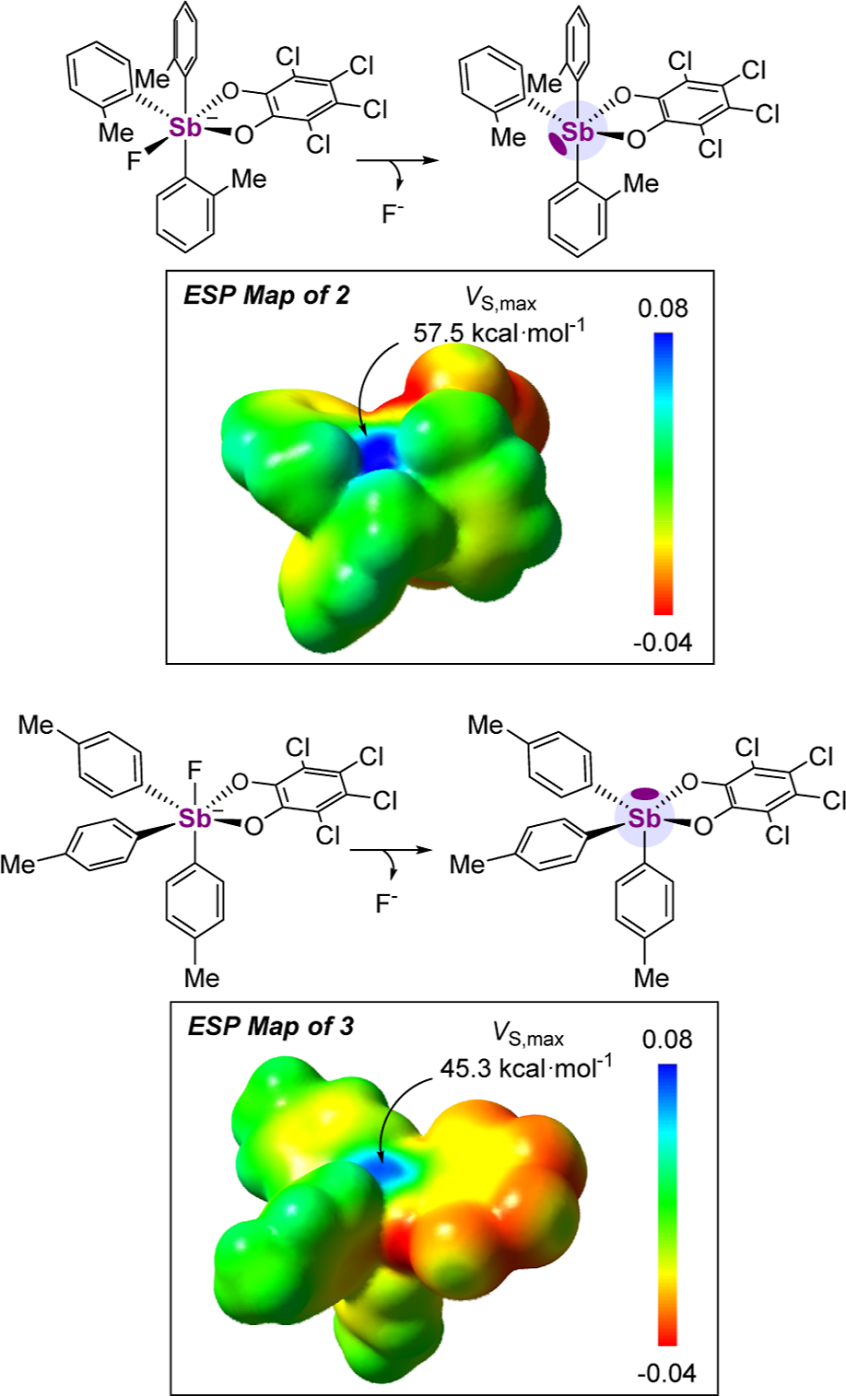
Computational removal of the bound fluoride from the optimized structures of [**2**-F]^−^ and [**3**-F]^−^ and subsequent ESP maps (isovalue: 0.001 a.u., gradient scale values given in a.u.).

We note that many of the reported computationally derived ESP maps of catecholatostiboranes analyze pseudo-SP geometry with a single σ hole,^22,25,28,37^ which do not appear to be the ground state geometries of these free receptors. However, upon removing the fluoride from the optimized structures of [**2**-F]^−^ and [**3**-F]^−^, we generate structures of **2** and **3** at a pseudo-SP geometry which bear a single σ hole on their ESP maps with corresponding *V*_S,max_ values of 57.5 and 45.3 kcal·mol^–1^, respectively (Figure 7). It would appear that at the computationally derived single σ hole-producing geometry, the dynamicity of potential anion binding is lost.

### Application of the Activation Strain Model

**Figure 8:**
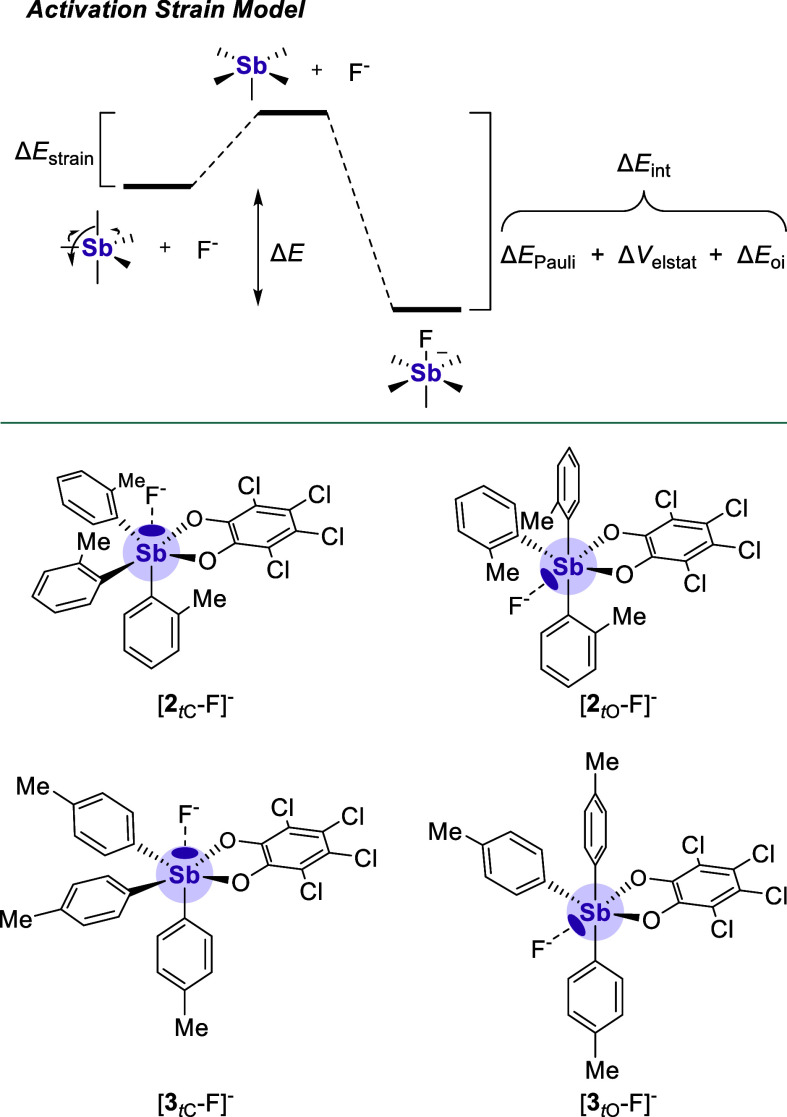
Top: visual representation of the activation strain model. Bottom: structures of the *t*O and *t*C isomers of [**2**-F]^−^ and [**3**-F]^−^.

Recognizing that the σ holes found in our analysis of **3** would allow for the two different binding geometries that we see between [**2**-F]^−^ and [**3**-F]^−^, we were keen to tease apart the components of each bonding situation. Thus, we computed two different isomers for [**2**-F]^−^ and [**3**-F]^−^ in which the fluoride is bound trans to the Sb–O_*o*-chloranil_ bond (*t*O) and trans to an Sb–C_aryl_ bond (*t*C), respectively (Figure 8). Complete details of these optimizations can be found in the Experimental Section. In each case, we identified stable minima for both potential isomers of [**2**-F]^−^ and [**3**-F]^−^. For [**2**-F]^−^, the *t*O isomer lies 3.1 kcal·mol^–1^ lower in energy than its *t*C isomer. As expected, this preference is inverted for [**3**-F]^−^ with its *t*C isomer lying 0.5 kcal·mol^–1^ lower in energy than its *t*O isomer.

We then sought to disentangle the energetic components that make up the F^–^ → Sb interaction of each isomer as a way to rationalize each receptor’s preference for a particular σ hole. Using Bickelhaupt’s activation strain model (Figure 8),^38^ we divided the total energy of a given F^–^ → Sb interaction (Δ*E*) into the energy associated with the deformation of the PnB donor to accept the fluoride (Δ*E*_strain_) and the energy associated with allowing the two species to interact (Δ*E*_int_). As tabulated in Table 1, Δ*E*_int_ was further parsed into electrostatic (Δ*E*_el_), orbital interaction (Δ*E*_oi_), and Pauli repulsion (Δ*E*_Pauli_) components using energy decomposition analysis (EDA) as implemented in the Amsterdam Density Functional (ADF) program. For both adducts, these calculations indicate that the *t*O isomer has a Δ*E*_int_ value that is ∼10–12 kcal·mol^–1^ greater in magnitude than that of the corresponding *t*C isomer. This arises from a more stabilizing Δ*E*_el_ and a less destabilizing Δ*E*_Pauli_. Indeed, this observation indicates that there is a deeper σ hole opposite to the Sb–O_*o*-chloranil_, which corroborates our ESP map findings and our previous interrogation of **1**.^13^

**Table 1:** EDA of the F^–^ → Sb Interactions with Values in kcal·mol^–1^^a^

compound	Δ*E*	Δ*E*_strain_	Δ*E*_int_	Δ*E*_oi_	Δ*E*_el_	Δ*E*_Pauli_
[**2**_*t*O_-F]^–^	–84.06	27.84	–111.90	–113.60	–181.40	183.70
[**2**_*t*C_-F]^–^	–81.55	20.48	–102.03	–114.96	–176.45	189.91
[**3**_*t*O_-F]^–^	–82.95	27.95	–110.90	–112.76	–174.70	177.06
[**3**_*t*C_-F]^–^	–82.98	16.36	–99.34	–112.59	–169.96	183.69
						
						

a Δ*E*_disp_ values were found to be inconsequentially small and can be found in the Supporting Information.

**Figure 9:**
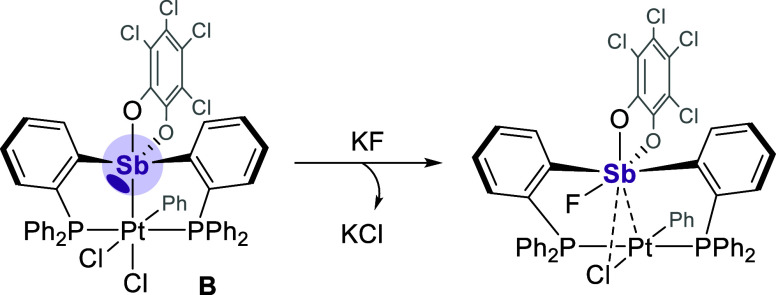
Preorganized σ hole found at the stiboranyl unit of **B**.

Yet, if this analysis is true, then why do we even observe *t*C isomers in the first place? Given that the *t*O isomers yield a more stabilizing Δ*E*_int_, the answer must lie in whether the Δ*E*_strain_ prevents adoption of the *t*O isomer. Indeed, the *t*C isomers of both [**2**-F]^−^ and [**3**-F]^−^ enjoy a lower Δ*E*_strain_ than their *t*O isomers. We find that while the Δ*E*_strain_ is similar for [**2**_*t*O_-F]^−^ and [**3**_*t*O_-F]^−^, this value is 4.1 kcal·mol^–1^ lower for [**3**_*t*C_-F]^−^ compared to [**2**_*t*C_-F]^−^. While [**3**_*t*C_-F]^−^ benefits from this lower Δ*E*_strain_, the potential benefit for [**2**-F]^−^ in trading increased stabilizing contributions for decreased destabilizing contributions is not observed, leading to the observed preference for the *t*O isomer with the fluoride bound at the deeper σ hole. A similar, more exaggerative example of such structural predisposition can be found with the stiboranyl unit of **B**(18) whose molecular geometry preorganizes the σ hole trans to a Sb–O_*o*-chloranil_ bond to be its preferred site of fluoride binding (Figure 9). For [**3**-F]^−^, binding at the shallower σ hole does lead to a less stabilizing Δ*E*_int_ but comes with an acceptable trade-off in the form of a less destabilizing Δ*E*_strain_. Thus, we contend that the single σ hole model is simply an oversimplification of the preferences in how these Lewis acids distort in order to accommodate an LB at a particular site and not the coalescing of shallower σ holes at a presupposed location.

We also note that the calculated fluoride binding energies seem to indicate that **2** is a stronger PnB donor than **3**, in opposition to our experimental findings. Indeed, the gas phase free energy of the fluoride exchange reaction between [**2**_*t*O_-F]^−^ and **3** is positive (1.41 kcal·mol^–1^), consistent with the greater fluoride ion affinity of **2**. However, when we include water solvation, the *in silico* reaction between [**2**_*t*O_-F]^−^ and **3** has a Δ*G*° of −6.36 kcal·mol^–1^ in favor of the formation of [**3**_*t*C_-F]^−^. Performing similar calculations that account for chloroform solvation still affords a negative Δ*G*° of −10.67 kcal·mol^–1^. We rationalize this occurrence by considering solvation effects. The *o*-Tol groups of [**2**_*t*O_-F]^−^ shield the fluoride anion, thus reducing the stabilization and the overall solvation of the antimonate anion. In contrast, [**3**_*t*C_-F]^−^ is more polar, enhancing solvation effects. These factors may serve to rationalize the higher observed Lewis acidity of **3** in solution.

## Conclusions

In summary, we describe an investigation into the Lewis acidities of two closely related catecholatostiboranes. Both compounds are capable of binding fluoride, though their preferred fluoride binding sites differ based on the steric pressure of their adorning methyl substituents. Moreover, we show *via* computations that a number of σ holes on the Sb(V) surface, and thus anion binding sites, are available to the fluoride anion. However, the preferred anion-binding geometries are not solely dependent upon the depth of a given σ hole, but rather the subtle interplay of minimizing strain energy while maximizing attractive anion–receptor interactions at a potential binding site. We also find that solvation must be considered to rationalize the experimentally observed fluoride anion affinity of these stiboranes in solution. We believe that these findings not only deepen our fundamental understanding of catecholatostiboranes but also can guide the development of new PnB-donor catalysts and anion transporters.

## Experimental Section

### General Methods

**2**(28) and tris(*p*-tolyl)stibine [(*p*-Tol)_3_Sb)]^39^ were synthesized according to literature procedures with spectra that matched those reported. 3,4,5,6-Tetrachloro-1,2-benzoquinone (*o*-chloranil) was purchased from Acros, tetrabutylammonium fluoride trihydrate (TBAF·3 H_2_O) was purchased from Apollo Scientific, and tetrabutylammonium difluorotriphenylsilicate (TBAT) was purchased from TCI America. These reagents were used without further purification. CHCl_3_ was dried over CaH_2_ and distilled prior to use. All other solvents used were ACS grade and used as received. NMR spectra were recorded using a Bruker Avance 500 NMR spectrometer (499.58 MHz for ^1^H, 125.63 MHz for ^13^C), Bruker Ascend 400 NMR spectrometer (376.56 MHz for ^19^F), or Bruker Ascend 500 NMR spectrometer (470.59 MHz for ^19^F). Chemical shifts (δ) are given in ppm and are referenced against residual solvent signals (^1^H, ^13^C) or external CF_3_CO_2_H (−76.55 ppm, ^19^F). Elemental analyses were performed at Atlantic Microlab (Norcross, GA). Spectrophotometric fluoride titrations were performed using a Shimadzu UV-2501 UV–vis spectrometer.

### Synthesis of [^*n*^Bu_4_N][2-F]

A solution of TBAT (42.0 mg, mmol, 7.8 × 10^–2^ mmol) in CH_2_Cl_2_ (2 mL) was added dropwise to a solution of **2** (50.0 mg, 7.8 × 10^–2^ mmol) in CH_2_Cl_2_ (3 mL). The vibrant yellow color of the solution faded to a pale color, and the resulting solution was allowed to stir for 15 min. Solvent was then removed in vacuo, and copious n-pentane (∼50 mL) was added to the resulting sticky substance which eventually solidified into an off-white powder. The solid was redissolved in CH_2_Cl_2_ (∼0.5 mL) and was layered with hexanes (15 mL). The product was fractionally recrystallized yielding clear colorless blocks. Yield: 10.1 mg (12%, 9.1 × 10^–3^ mmol). Single crystals suitable for X-ray crystallography were also obtained as clear, colorless blocks *via* diffusion of hexanes into a CDCl_3_ solution of the compound. ^1^H NMR in CDCl_3_ is included as Figure S1. ^19^F NMR (377 MHz, CDCl_3_): δ −69.17 (s), −108.15 (broad s), −114.57 (broad s). Elemental Anal. Calcd for C_43_H_57_Cl_4_FNO_2_Sb: C, 57.23; H, 6.37; N, 1.55. Found: C, 56.52; H, 6.42; N, 1.54.

### Synthesis of **3**

To a solution of (*p*-Tol)_3_Sb (0.25 g, 0.63 mmol) in CH_2_Cl_2_ (3 mL) was added *o*-chloranil (0.16 g, 0.63 mmol) in one portion. The resulting solution quickly changed from a deep red to a yellow color indicating the oxidation of the antimony center. This solution was allowed to stir for 5 min, after which hexanes (∼5 mL) was added to afford a yellow precipitate. This precipitate was collected by filtration and washed with hexanes (3 × 10 mL). Yield: 0.33 g (83%, 0.52 mmol). Single crystals suitable for X-ray crystallography were obtained as yellow blocks *via* evaporation of a CH_2_Cl_2_ solution of the compound. ^1^H NMR (500 MHz, CDCl_3_): δ 7.68 (d, *J* = 8.1 Hz, 6H, SbAr), 7.32 (d, *J* = 7.9 Hz, 6H, SbAr), 2.41 (s, 9H, SbAr–CH_3_). ^13^C NMR (126 MHz, CDCl_3_): δ 144.47 (s), 142.59 (s), 135.35 (s), 132.49 (s), 130.50 (s), 120.46 (s), 116.60 (s), 21.72 (s). Elemental Anal. Calcd for C_27_H_21_Cl_4_O_2_Sb: C, 50.59; H, 3.30. Found: C, 50.64; H, 3.12.

### Synthesis of [^*n*^Bu_4_N][3-F]

A solution of TBAT (42.0 mg, 7.8 × 10^–2^ mmol) in CH_2_Cl_2_ (2 mL) was added dropwise to a solution of **3** (50.0 mg, 7.8 × 10^–2^ mmol) in CH_2_Cl_2_ (3 mL). The vibrant yellow color of the solution quickly faded, and the resulting solution was allowed to stir for 15 min. Solvent was then removed in vacuo, and copious *n*-pentane (∼50 mL) was added to the resulting sticky substance which eventually solidified into a white powder. Yield: 60.6 mg (86%, 6.7 × 10^–2^ mmol). Single crystals suitable for X-ray crystallography were obtained as clear, colorless blocks *via* layering of hexanes onto a CH_2_Cl_2_ solution of the compound. ^1^H NMR (500 MHz, CDCl_3_): δ 7.74 (d, *J* = 7.5 Hz, 4H, equatorial SbAr), 7.30 (d, *J* = 7.7 Hz, 2H, axial SbAr), 7.13 (d, *J* = 7.5 Hz, 4H, equatorial SbAr), 6.98 (d, *J* = 7.7 Hz, 2H, axial SbAr), 2.86 (m, 8H, ^*n*^Bu_4_N), 2.33 (s, 6H, equatorial SbAr–CH_3_), 2.24 (s, 3H, axial SbAr–CH_3_), 1.36 (m, 8H, ^*n*^Bu_4_N), 1.19 (m, 8H, ^*n*^Bu_4_N), 0.90 (t, *J* = 7.2 Hz, 12H, ^*n*^Bu_4_N). ^13^C NMR (126 MHz, CDCl_3_): δ 149.17 (s), 143.79 (s), 143.55 (s), 138.05 (s), 137.70 (s), 135.30 (s), 134.26 (s), 128.73 (s), 128.62 (s), 116.71 (s), 115.14 (s), 58.33 (s), 24.01 (s) 21.57 (s), 21.46 (s), 19.72 (s), 13.84 (s). ^19^F NMR (471 MHz, CDCl_3_): δ −83.63. Elemental Anal. Calcd for C_43_H_57_Cl_4_FNO_2_Sb: C, 57.23; H, 6.37; N, 1.55. Found: C, 57.22; H, 6.51; N, 1.55.

### Crystallography

All crystallographic measurements were performed at 110(1) K using a Bruker D8 QUEST diffractometer (graphite monochromated Mo Kα radiation, λ = 0.71073 Å). In each case, a specimen of suitable size and quality was selected and mounted onto a nylon loop. Integrated intensity information for each reflection was obtained by reducing the data frames using APEX3.^40^ The semiempirical method SADABS was used for the absorption corrections.^41^ The structures were solved by direct methods using ShelXT^42^ and refined against *F*^2^ with anisotropic temperature-dependent parameters for all non-hydrogen atoms using ShelXL^43^ using Olex2.^44^ All H atoms were geometrically placed and refined using a riding model. Diamond4 was used for final data presentation. The structural data has been deposited with the Cambridge Structural Database. Positional disorder was found to affect two of the *o*-Tol rings of [**2**-F][^*n*^Bu_4_N]. This disorder was modeled, leading to an improvement in the quality of the refinement. The structure shown in Figure 2 of the main text is the major component.

### Spectrophotometric Fluoride Titrations

A CHCl_3_ solution of either **2** (1.06 × 10^–4^ M) or 3 (1.02 × 10^–4^ M) (3030 μL) was prepared in a quartz cuvette with a small stir bar. Aliquots of an CHCl_3_ solution of TBAF·3 H_2_O (1.85 × 10^–2^ M) were added incrementally. After each addition, the solution was stirred for at least 30 s, and a UV–vis spectrum was recorded at room temperature. The resulting absorbance data were fitted to the calculated absorbance values with a 1:1 binding isotherm to provide the fluoride binding constant. The fitting was carried out by hand using Microsoft Excel. Initial reactions of fluoride with residual acid present in CHCl_3_ are corrected for.

### ^19^F NMR Fluoride Competition Experiment

An equivalent of [^*n*^Bu_4_N][**2**-F] was dissolved in benchtop CDCl_3_ and an initial ^19^F NMR spectrum was taken. To this NMR tube, 0.5 equiv of **3** dissolved in CDCl_3_ was added and the tube was inverted several times to ensure mixing. A ^19^F NMR spectrum was then taken, and this process was repeated such that 1.0 equiv of **3** was added to the solution.

### Geometry Optimizations

For gas-phase calculations: geometry optimizations were performed in Orca 5.0.2^45−47^ using PBEh-3c/def2-mSVP^48^ with the default defgrid2 settings. Some optimizations were done with the VeryTightSCF and VeryTightOPT keywords to resolve issues in finding a minimum-energy geometry. Frequency calculations were performed at the same level of theory to verify that all optimized geometries were at a local minimum on the potential energy surface. Where possible, geometries were optimized from the initial crystal structure.^28^ To generate [**2**_*t*C_-F]^−^ and [**3**_*t*O_-F]^−^, a fluoride was added to the previously optimized, free Lewis acid trans to an aryl carbon and trans to an oxygen, respectively, in Avogadro^49^ and the resulting fluoroantimonate was optimized. To ensure the lowest-energy conformation of [**2**-F]^−^ was achieved, we optimized geometries in which the methyl groups of all three *o*-Tol groups pointed up toward the F^–^ (uuu), two methyl groups pointed up with one down (uud), one methyl group up with two down (udd), and all three pointing down and away from the F^–^ (ddd). We optimized local minima for each of these conformations, but the uuu geometry was the lowest in energy, so we used it for our analyses since we expect it to be an attainable static structure throughout the molecule’s dynamic changes. Single-point energies were calculated at the optimized geometries in Orca using the DSD-BLYP functional^50^ with Grimme’s empirical D3 correction using the Becke–Johnson (BJ) damping function^51^ with the def2-QZVPP basis set using the RIJCOSX approximation. For water-phase calculations: geometry optimizations were performed in Orca 5.0.2 using PBEh-3c/def2-mSVP with the default defgrid2 settings and the SMD solvation model with water.^52^ Some optimizations were done with the VeryTightSCF and VeryTightOPT keywords to resolve issues in finding a minimum-energy geometry. The solvated geometries were optimized starting from the gas-phase optimized geometries. Frequency calculations were performed at the same level of theory (including SMD solvation) to verify that all optimized structures were at a local minimum on the potential energy surface. Single-point energies were calculated at the solvated geometries in Orca using the DSD-BLYP functional^50^ with Grimme’s empirical D3 correction using the Becke–Johnson (BJ) damping function^51^ with the def2-QZVPP basis set using the RIJCOSX approximation in addition to the SMD solvation model.^53^ The necessary corrections were added to these single-point energies from the frequency calculations done at the PBEh-3c/def-mSVP level to obtain enthalpy and Gibbs free energy values for each solvated species.

### Electrostatic Potential Map Analysis

The structures of **2** and **3** optimized in Orca were used as inputs for single-point energy calculations conducted in Gaussian 16 using the B3LYP functional and the following mixed basis sets: aug-cc-pVTZ-PP^54−56^ for Sb & 6-311+g(2d,p) for all other atoms. In each case, an ESP map was determined at an isodensity value of 0.001 electrons/bohr^3^. ESP maps were visualized in GaussView and Multiwfn was used to identify areas of maximum electrostatic potential (*V*_S,max_). In another case, the fluoride anions of the gas-phase structures of [**2**_*t*O_-F]^−^ and [**3**_*t*C_-F]^−^ optimized in Orca, which correspond to the preferential geometries of the fluoroantimonates in the solid-state, were removed and the resulting structures were subjected to single-point calculations using the same parameters. Their ESP maps were generated and examined similarly to the free receptors.

### Activation Strain Analysis and Energy Decomposition Analysis

For the activation strain model analysis, the structures optimized in Orca were used as inputs for single-point energy calculations and energy decomposition analysis (EDA)^57−59^ computations conducted in ADF 2022.101^60^ using the M06 functional^,61,62^ paired with the D3 model to account for dispersion effects (Δ*E*_disp_).^63^ The QZ4P basis set^64^ as implemented in the ADF program was used without frozen-core approximation and with good numerical quality. The zeroth-order regular approximation (ZORA) Hamiltonian was employed to account for scalar relativistic effects.^65^ To avoid numerical issues, the “Fix Dependencies” function in ADF was enabled. Δ*E*_strain_ was determined by subtracting the single-point energy of the free Lewis acid from the single-point energy of the strained Lewis acid with no F^–^ bound. EDA as implemented in ADF directly provided Δ*E*_el_, Δ*E*_oi_, and Δ*E*_Pauli_.

### Fluoride Ion Affinity Calculations

The gas-phase fluoride ion affinity (FIA) values of **2** and **3** were derived from a known procedure implementing the isodesmic reaction of trimethylsilyl fluoride with a Lewis acid.^66^ The structures optimized in Orca were used as inputs for single-point energy calculations using the DSD-BLYP functional^50^ with Grimme’s empirical D3 correction using the Becke–Johnson damping function with the def2-QZVPP basis set^67^ using the RIJCOSX approximation. The necessary corrections were added to these single point energies from the analytical frequency calculations done at the PBEh-3c/def2-mSVP level^48^ to obtain the enthalpy values for each species. This reaction was benchmarked at the CCSD(T)/CBS level to the known FIA of trimethylsilylium of 952.5 kJ·mol^–1^.^68^

## Supporting Information Available

Experimental NMR spectra, complete data table, and optimized structures in XYZ format (PDF)
