# Supplementary material for: Steric Selection of Anion Binding Sites by Organoantimony(V) Pnictogen Bond Donors: An Experimental and Computational Study
Source: Inorg Chem. 2024 Dec 3;63(50):23568–76. doi: 10.1021/acs.inorgchem.4c03178 (PMC11653240; doi:10.1021/acs.inorgchem.4c03178)
Supplement: Supplementary file 1 — ic4c03178_si_001.pdf [file ic4c03178_si_001.pdf]

Steric Selection of Anion Binding Sites by Organoantimony(V) Pnictogen Bond Donors: An Experimental and Computational Study

Brendan L. Murphy, Logan T. Maltz, and François P. Gabbaï*

Department of Chemistry, Texas A&M University, College Station, Texas 77843-3255, United States

* Corresponding author email: francois@tamu.edu

Supporting Information

This PDF file includes

Contents:

Figure S1. ^{1}H NMR spectrum of [^{n}Bu$_4$N][**2**-F] in $CDCl_3$.

Figure S2. ^{19}F NMR spectrum of [^{n}Bu$_4$N][**2**-F] in $CDCl_3$.

Figure S3. ^{1}H NMR spectrum of **3** in $CDCl_3$.

Figure S4. ^{13}C NMR spectrum of **3** in $CDCl_3$.

Figure S5. ^{1}H NMR spectrum of [^{n}Bu$_4$N][**3**-F] in $CDCl_3$.

Figure S6. ^{19}F NMR spectrum of [^{n}Bu$_4$N][**3**-F] in $CDCl_3$.

Figure S7. ^{13}C NMR spectrum of [^{n}Bu$_4$N][**3**-F] in $CDCl_3$.

Figure S8. ^{19}F NMR spectra showing the challenging of [**3**-F]$^{-}$ with one equivalent of **2**.

Table S1. Full output of energy decomposition analysis results.

Table S2. TMS-anchored fluoride competition experiments.

Chart S1. Comparison of the energy decomposition analyses of [**2*t*$_O$-F**]$^{-}$ and [**3*t*$_C$-F**]$^{-}$.

Chart S2. Comparison of the energy decomposition analyses of [**2*t*$_O$-F**]$^{-}$ and [**2*t*$_C$-F**]$^{-}$.

Chart S3. Comparison of the energy decomposition analyses of [**3*t*$_C$-F**]$^{-}$ and [**3*t*$_O$-F**]$^{-}$.

Table S3. XYZ coordinates of the optimized geometries.

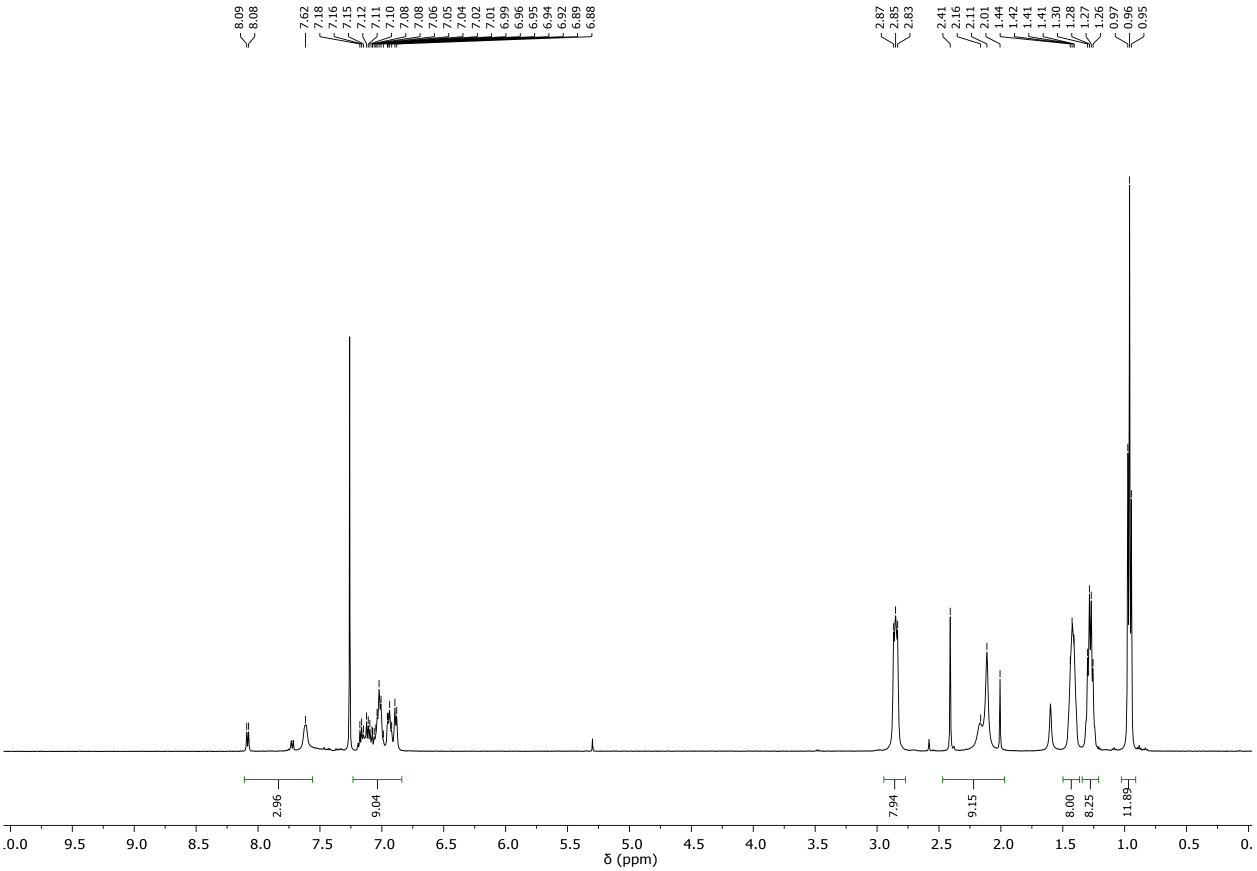

Figure S1. ^{1}H NMR ($CDCl_3$, 500 MHz) spectrum of [^{n}Bu$_4$N][**2**-F]. While the origins of each peak not associated with the [^{n}Bu$_4$N]$^{+}$ counterion cannot be elucidated here, we note a 4:3 ratio of aromatic to aliphatic resonances that correspond to those expected on the *o*-Tol rings of [^{n}Bu$_4$N][**2**-F].

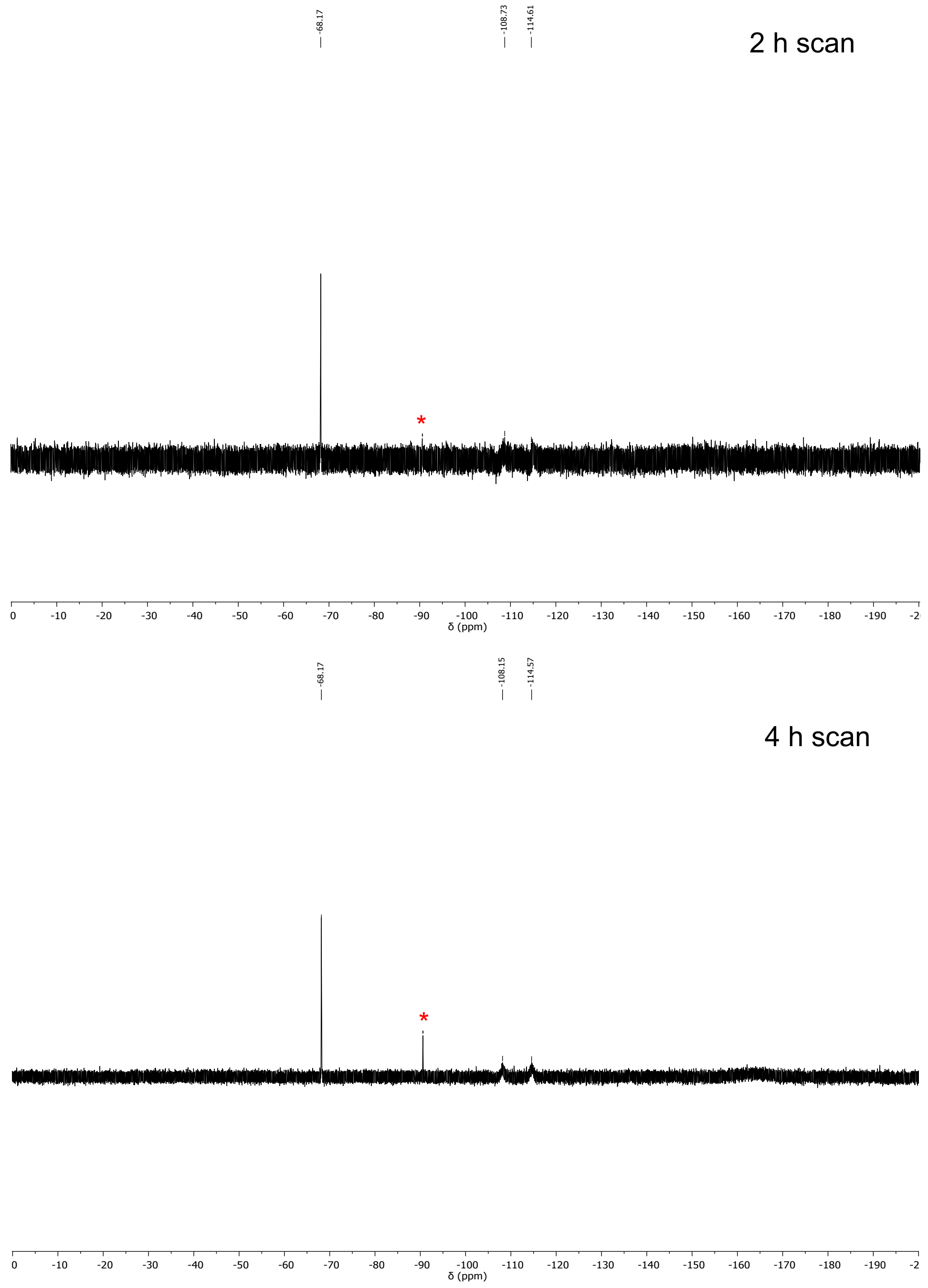

Figure S2. ^{19}F NMR ($CDCl_3$, 471 MHz) spectrum of [$^{n}Bu_4N$][**2**-F]. The poor solubility of crystals required extended acquisition times, during which time an impurity (*) appears and grows in intensity.

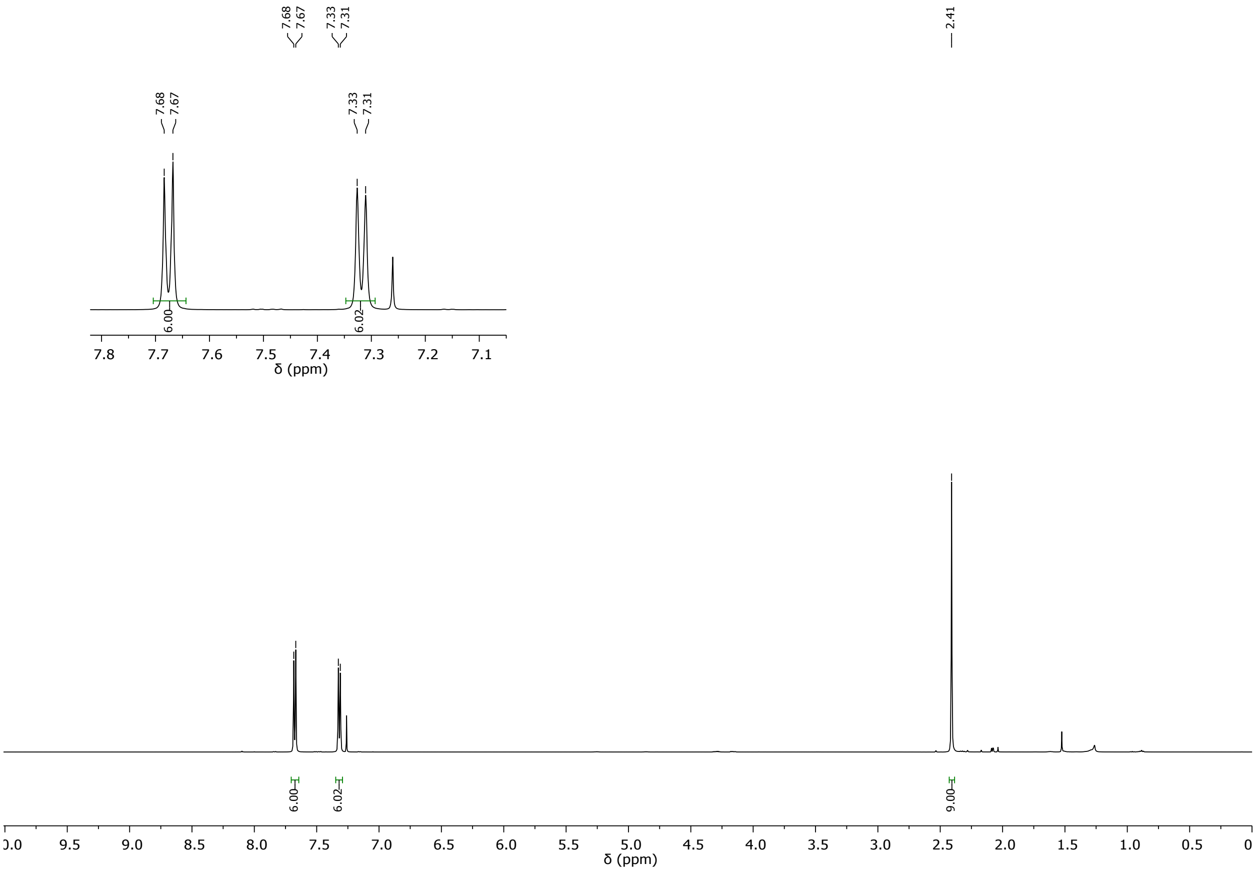

Figure S3. ^{1}H NMR ($CDCl_3$, 500 MHz) spectrum of **3**.

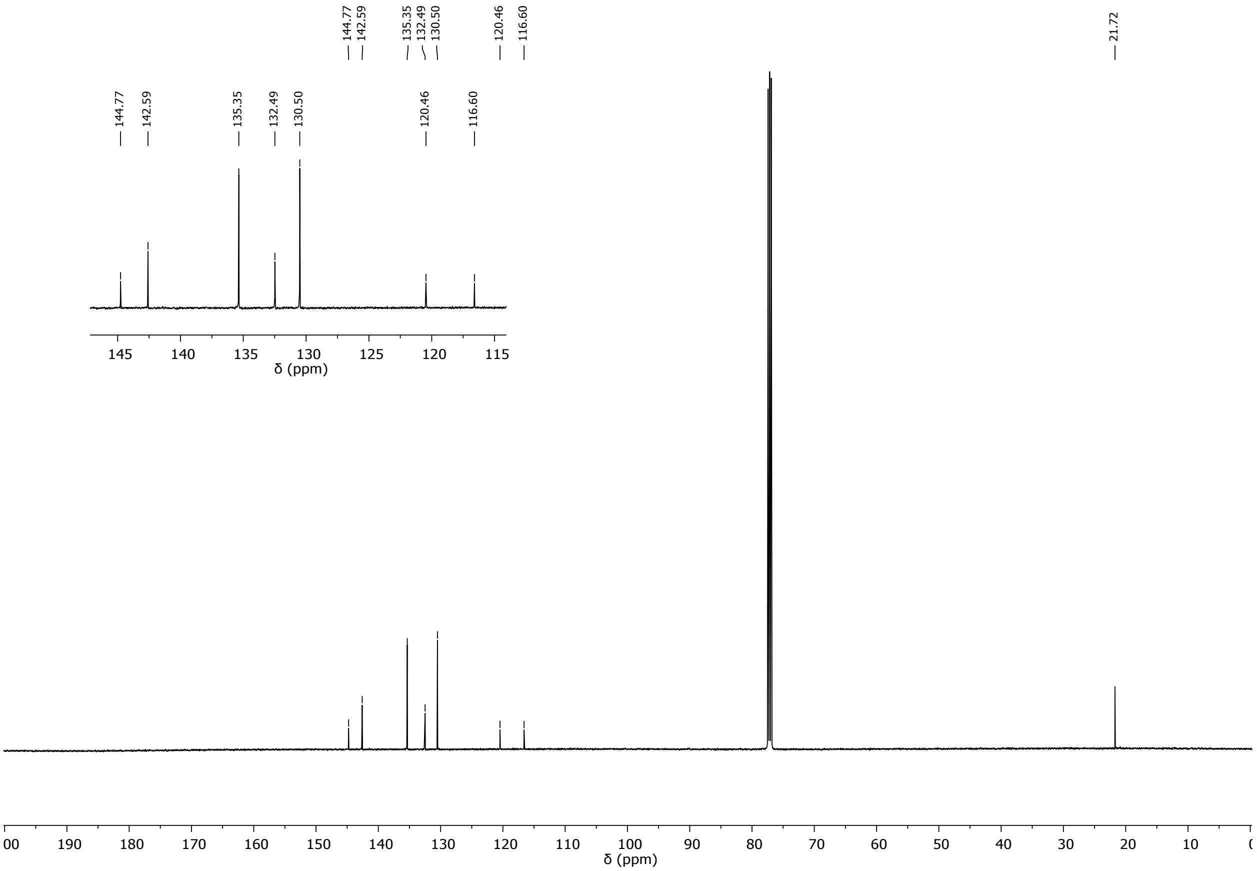

Figure S4. ^{13}C NMR ($CDCl_3$, 126 MHz) spectrum of **3**.

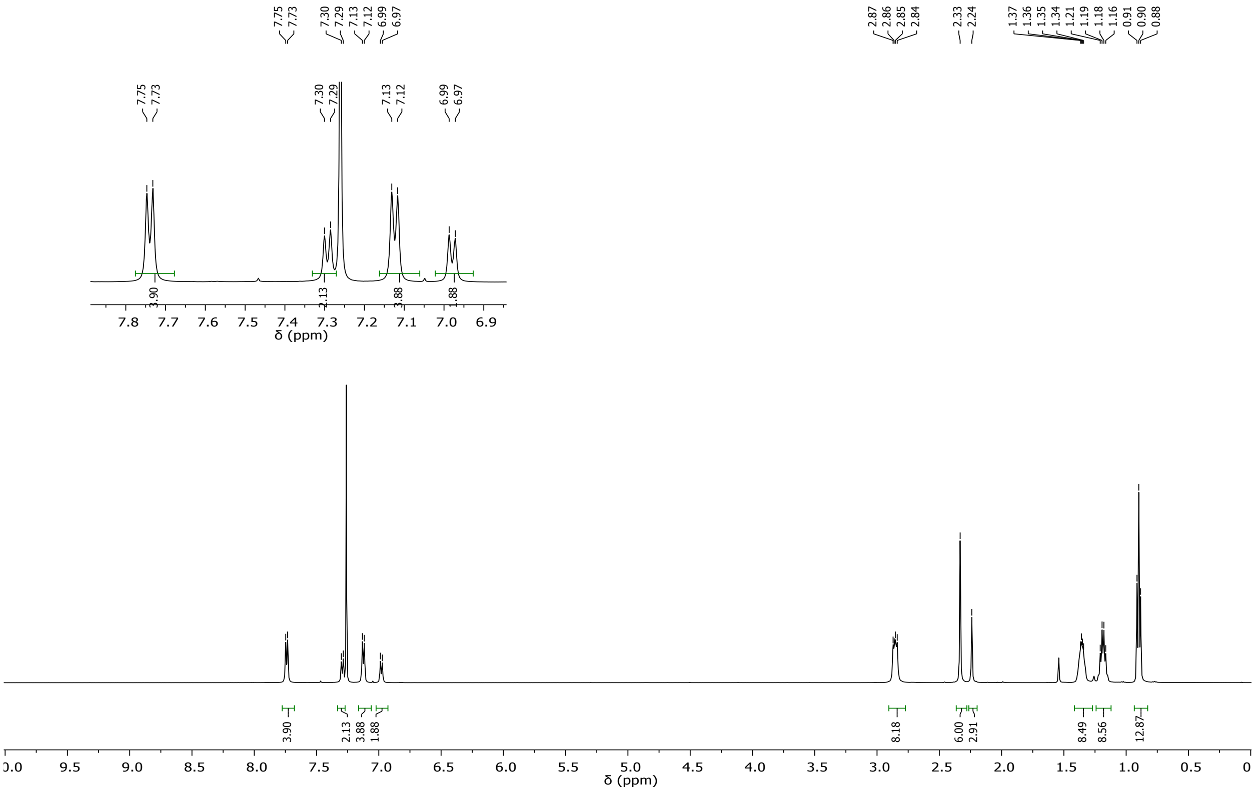

Figure S5. ^{1}H NMR ($CDCl_3$, 500 MHz) spectrum of [$^{n}Bu_4N$][**3**-F].

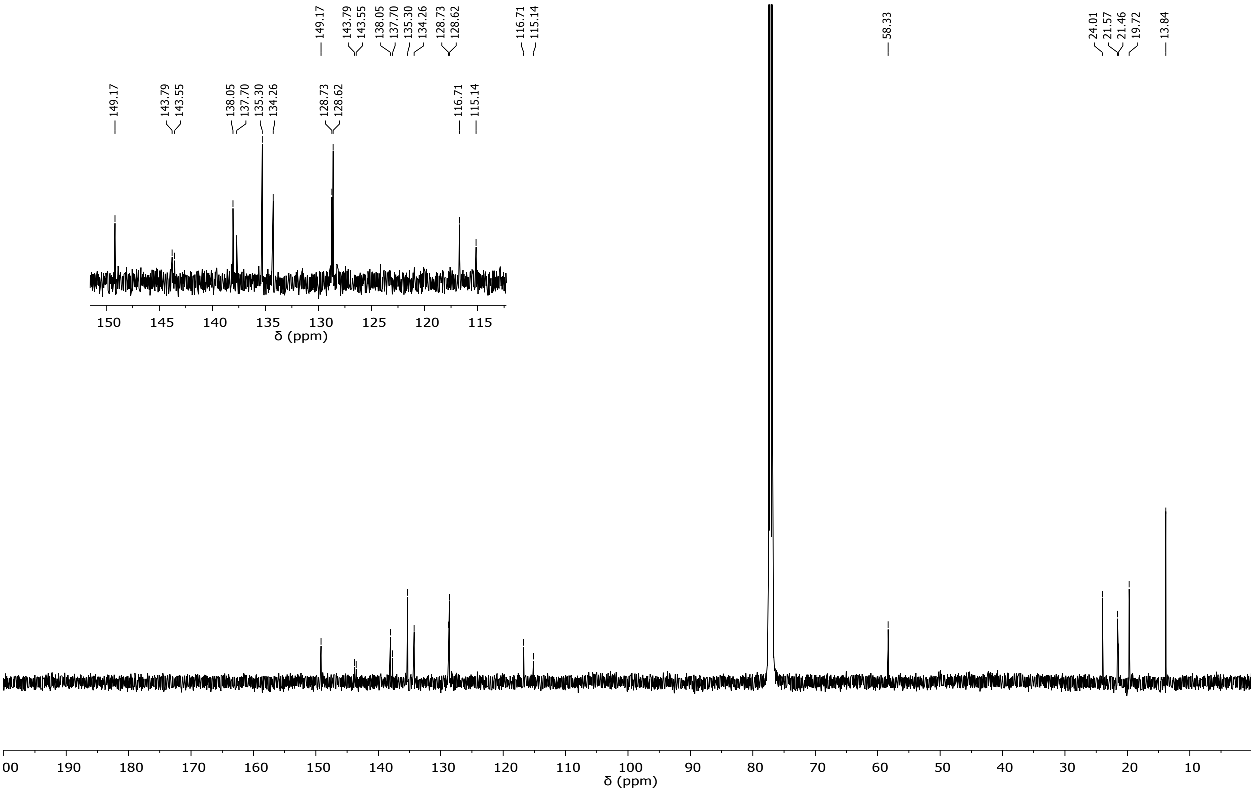

Figure S6. ^{13}C NMR ($CDCl_3$, 126 MHz) spectrum of [$^{n}Bu_4N$][**3**-F]. Solvent peak is truncated for clarity.

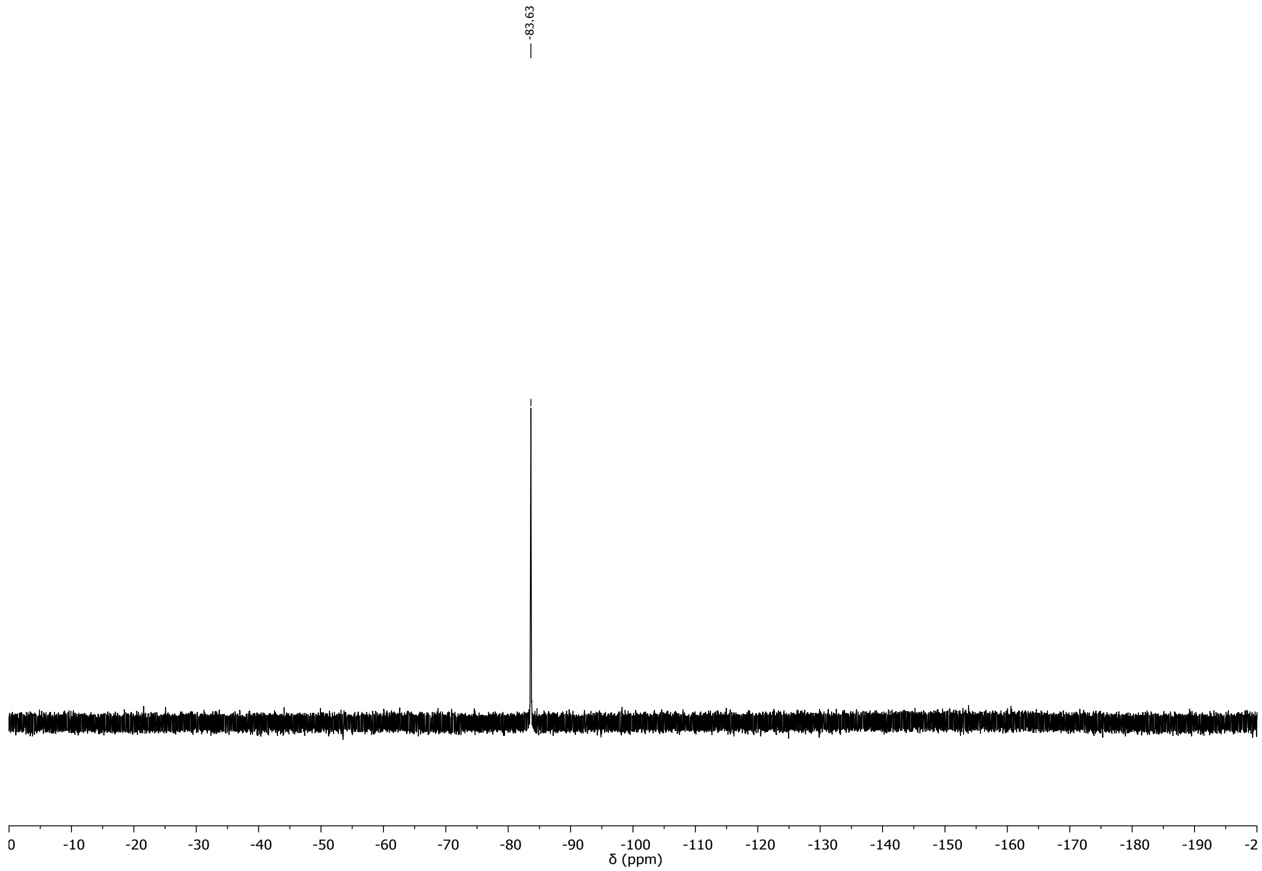

Figure S7. ^{19}F NMR ($CDCl_3$, 471 MHz) spectrum of [$^{n}Bu_4N$][**3**-F].

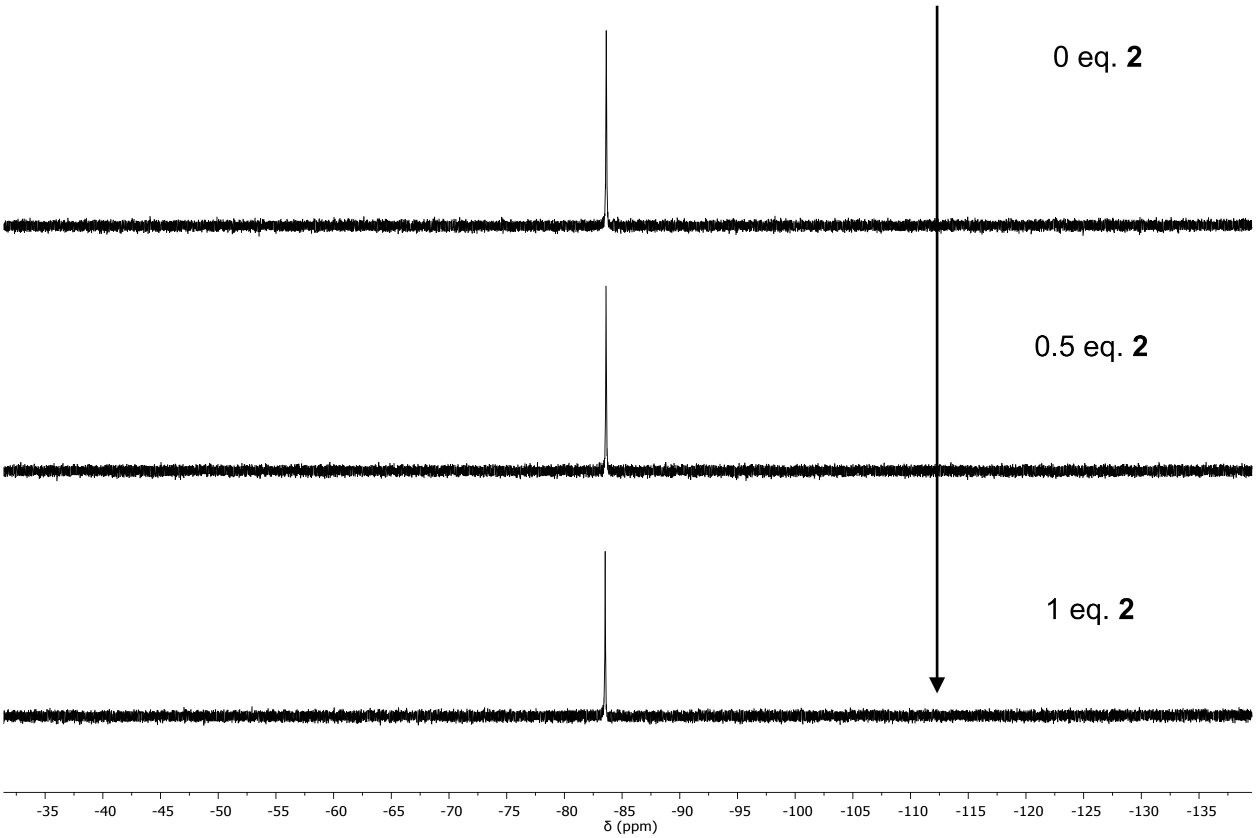

Figure S8. ^{19}F NMR ($CDCl_3$, 471 MHz) spectra of challenging [^{n}Bu$_4$N][**3**-F] with increments of **2**. No peaks associated with [**2**-F]$^-$ were detected.

Table S1. Full output from energy decomposition analyses. Values given are in kcal·mol^{-1}.

	[**2**$_{tO}$-**F**]$^-$	[**2**$_{tC}$-**F**]$^-$	[**3**$_{tO}$-**F**]$^-$	[**3**$_{tC}$-**F**]$^-$
Fluoride ion affinity	82.56	79.21	81.05	80.89
Total Energy (ΔE)	-84.06	-81.55	-82.95	-82.98
Strain Energy (ΔE_{strain})	27.84	20.48	27.95	16.36
Interaction Energy (ΔE_{int})	-111.90	-102.03	-110.90	-99.34
Dispersion Energy	-0.60	-0.53	-0.50	-0.48
Orbital Interaction Energy (ΔE_{oi})	-113.60	-114.96	-112.76	-112.59
Electrostatic Energy (ΔE_{el})	-181.40	-176.45	-174.70	-169.96
Pauli repulsion (ΔE_{Pauli})	183.70	189.91	117.06	183.69

Table S2. TMS-anchored fluoride competition experiments. Values are given in kJ·mol^{-1}.

	**2**	**3**	[**2**$_{tO}$-**F**]$^-$	[**3**$_{tC}$-**F**]$^-$
G_{gas}	-8587525.75	-8587540.69	-8849951.56	-8849960.58
H_{gas}	-8587280.96	-8587285.98	-8849701.35	-8849700.07
G_{water}	-8587562.21	-8587568.73	-8850095.94	-8850129.08
H_{water}	-8587319.90	-8587315.60	-8849848.23	-8849871.41
$G_{chloroform}$	-8587653.25	-8587631.81	-8850137.20	-8850160.39
$H_{chloroform}$	-8587410.21	-8587386.34	-8849888.52	-8849907.90

Chart S1. Comparison of the energy decomposition analyses of [**2***t*$_O$**-F**]$^-$ (orange) and [**3***t*$_C$**-F**]$^-$ (gray).

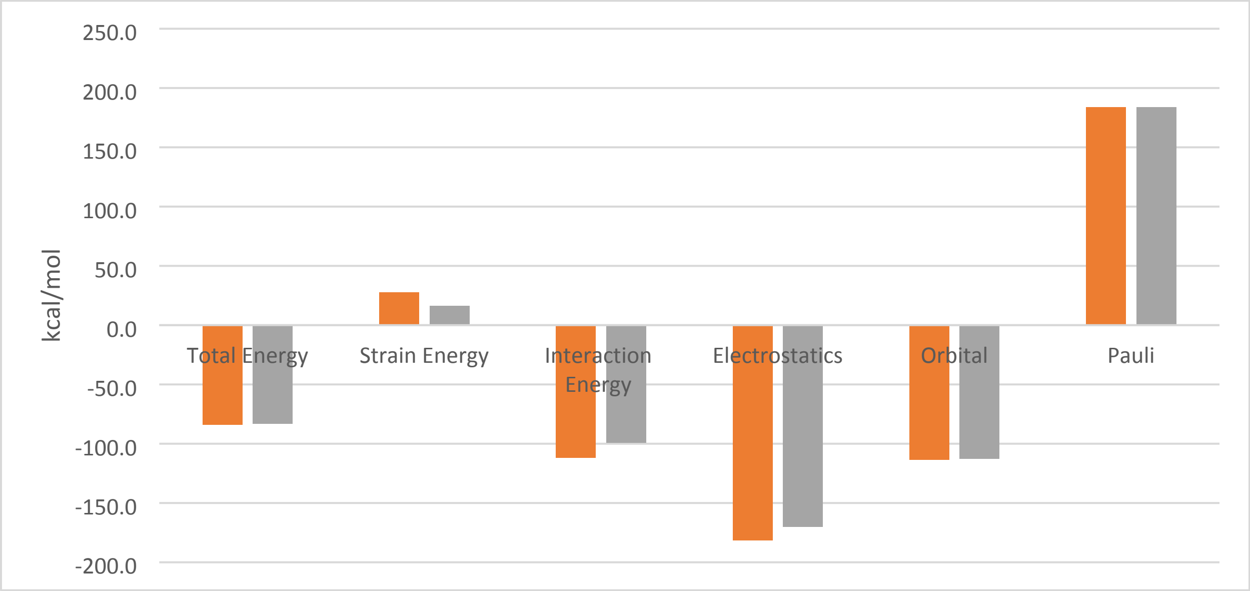

Chart S2. Comparison of the energy decomposition analyses of [**2***t*$_O$**-F**]$^-$ (orange) and [**2***t*$_C$**-F**]$^-$ (blue).

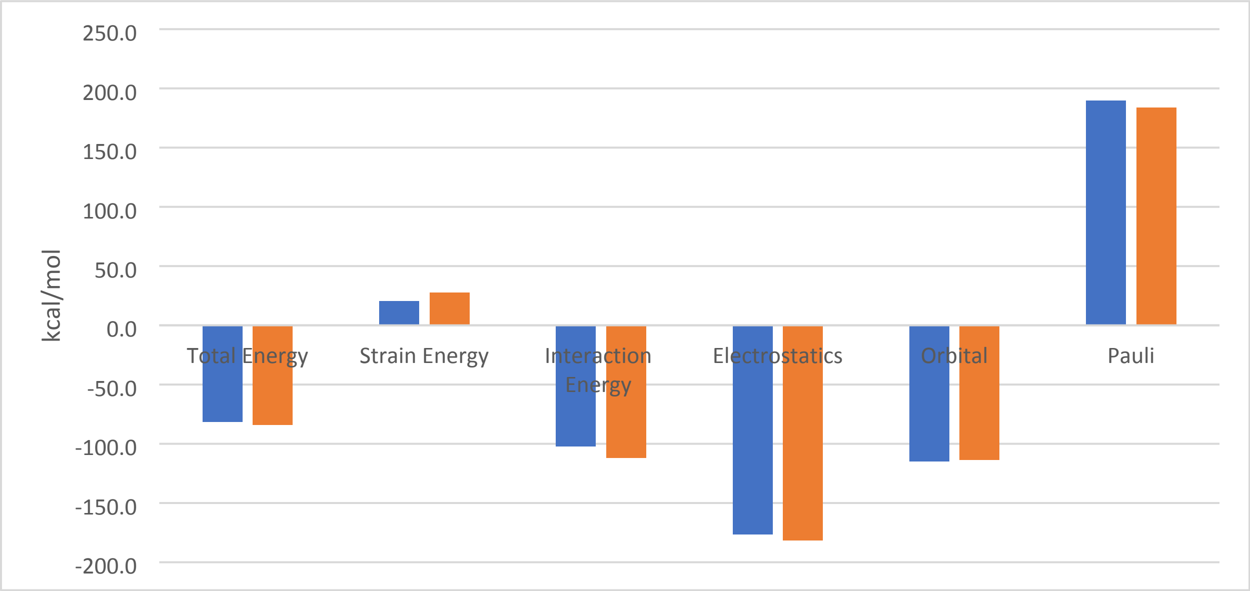

Chart S3. Comparison of the energy decomposition analyses of [**3t_C-F**]$^-$ (gray) and [**3t_O-F**]$^-$ (yellow).

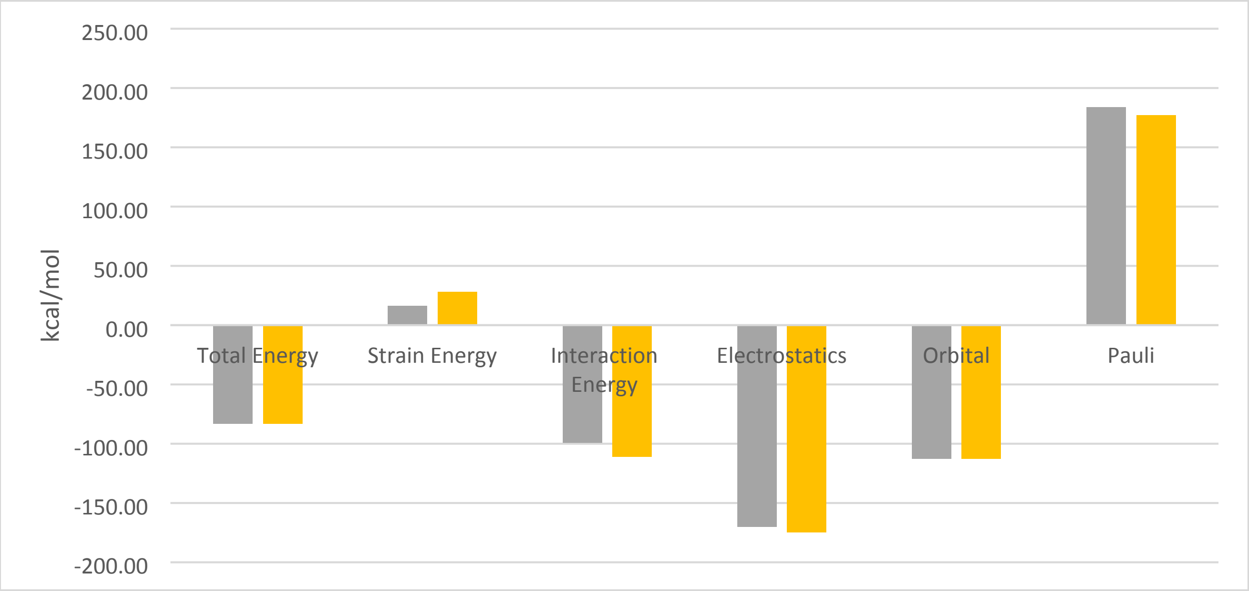

Table S3. XYZ coordinates of the optimized geometries.

2 (gas phase)

C	1.76674462418601	-0.68623400649754	0.03645246917880
C	3.01312473500654	-1.27054436970397	0.19214250452156
C	4.16512995049072	-0.48028020238018	0.14897969740462
C	4.06451835732232	0.89297339763289	-0.03590584353388
C	2.80707146588565	1.48560284473860	-0.17969227942351
C	1.67231199663060	0.69928247562089	-0.15057353266512
C	-2.31532737239482	1.52066266199141	-0.60064846384232
C	-2.30314776550109	2.75428395233052	0.06582293195080
C	-3.15633327365289	3.75210364105878	-0.39939621751328
H	-3.16480910790359	4.70957229621537	0.10748505324966
C	-3.99004866800591	3.55420715351080	-1.48918216988040
H	-4.63593505413985	4.35494846828269	-1.82483109001101
C	-3.99769141809030	2.33192574930967	-2.13660782030565
H	-4.64676421540880	2.15949384154965	-2.98466980567908
C	-3.16155444017050	1.32106884065748	-1.68583332048601
H	-3.19152434673165	0.36405162420221	-2.19287253044082
C	-1.43222410723395	3.05050824318402	1.25376839373033
H	-1.42947790804715	2.24017070121814	1.98413130712059
H	-1.78106590941607	3.94307782917426	1.77129964508477
H	-0.39972732137471	3.22327433984413	0.95252207198126
C	-1.84576547646687	-1.62878509357754	-1.32248270138939
C	-1.44697321398936	-1.68317022300746	-2.66063830928520
C	-1.99339443642937	-2.68114849991561	-3.46017516337062
H	-1.70273772649296	-2.73989308311430	-4.50187814648838
C	-2.89265987080589	-3.60499973038456	-2.94812996793938
H	-3.29880400397392	-4.37250686542513	-3.59358971133346
C	-3.25934296192435	-3.55272156969372	-1.61461624189595
H	-3.94688853813588	-4.28021770835518	-1.20477341355193
C	-2.73184794795326	-2.56079321741202	-0.79957846295651
H	-3.00815876980401	-2.53904845539315	0.24830268801045
C	-0.44578583323150	-0.71812528984034	-3.22557853316104
H	-0.72932562626664	0.32418424282607	-3.06119826105696
H	-0.33375789791198	-0.84728471206388	-4.30075806825963
H	0.53957423652776	-0.86934137153741	-2.78064452783027
C	-1.56599644007240	-0.53001739652878	1.99877460765827
C	-2.86835830459342	-0.37811992222329	2.49566056769427
C	-3.07158410203040	-0.59973644503818	3.85609932265351
H	-4.07235939356948	-0.49570241518288	4.25725429813600
C	-2.03262896078979	-0.95013924393509	4.70195267997886
H	-2.22685880185608	-1.11041646257858	5.75425868909767
C	-0.75348611896409	-1.10532217686856	4.19606563046422
H	0.06468296285618	-1.39194810937216	4.84255270346117
C	-0.52375962991624	-0.90224120581226	2.84505611428227
H	0.47480168054518	-1.05423396321934	2.46175418695158
C	-4.05207948921992	0.01183102218616	1.65538860862015
H	-4.08341310229845	1.08669193695047	1.47485234333665
H	-4.97873197537871	-0.26075549731740	2.15873741041269
H	-4.06856097445492	-0.47266267983755	0.67937607679129
Cl	3.09806807544584	-2.96415738268175	0.43741037095523
Cl	5.70234518262117	-1.21505415711137	0.33087516802281
Cl	5.47247090467680	1.86783688004352	-0.08853510816642
Cl	2.63308418370644	3.17837316255509	-0.38932760478698
O	0.62675973933982	-1.34878417755865	0.06460043437171
O	0.44388054826572	1.18974372226903	-0.28937113069053
Sb	-1.07083830890559	-0.13420585378407	-0.02718344917744

2 (water phase)

C	1.71240521038717	-0.74562792518053	0.03003298418099
C	2.95943287941106	-1.33780638836476	0.15205086861912
C	4.12154331432932	-0.56363442217836	0.08183990287281

C	4.03361877122508	0.80774602542492	-0.10112795410665
C	2.77761301244376	1.41287370967029	-0.21329747772044
C	1.63188385017182	0.64588644573790	-0.15189419933127
C	-2.35118555903859	1.55629905899106	-0.58824014143189
C	-2.35034911732734	2.78424483959959	0.08942886052524
C	-3.17587001000476	3.79714986579203	-0.39842499173677
H	-3.19446935861655	4.74918185048527	0.11853705282996
C	-3.97324931063036	3.61608291059707	-1.51891342948021
H	-4.60016106358140	4.42571057022580	-1.86943877809147
C	-3.96936354444718	2.39797379592250	-2.17789608594995
H	-4.58958951176921	2.23883557190623	-3.04995684931822
C	-3.15931204963442	1.37433505196710	-1.70660095101593
H	-3.17552906623736	0.42445925164723	-2.22691839245879
C	-1.51951342549710	3.06982569776353	1.30713218772600
H	-1.50062648674186	2.23727056698097	2.01016050377516
H	-1.91193935351044	3.93417421298115	1.84096263013347
H	-0.48616316994595	3.29641141288660	1.04264461882457
C	-1.86031134253121	-1.62226414478753	-1.34567327216615
C	-1.39457683005752	-1.68627951899424	-2.66304286093852
C	-1.88405681137955	-2.70934320233282	-3.47110631764982
H	-1.54436779691724	-2.77543984799680	-4.49748941152235
C	-2.79211622319607	-3.64157678165156	-2.98818360393687
H	-3.15329992020388	-4.42535860967882	-3.64109290525008
C	-3.23046454286904	-3.57174650855310	-1.67579257023257
H	-3.93213008908890	-4.29917216371900	-1.29006264939696
C	-2.76060650167658	-2.55798561217634	-0.85104577654090
H	-3.09977545443772	-2.51194052569215	0.17703754430151
C	-0.39979257113625	-0.70663998529614	-3.20999480268279
H	-0.72570326993836	0.32711419687600	-3.07415429593074
H	-0.24754305273752	-0.85818677759426	-4.27730602963970
H	0.57373124705230	-0.81290003339029	-2.72695582767075
C	-1.56957290362968	-0.49967449985226	2.03770086679429
C	-2.87167763157700	-0.40354182644986	2.54571294279783
C	-3.05080939428420	-0.62790248409712	3.91077138582667
H	-4.05187545998829	-0.57151328095976	4.32044607569775
C	-1.98529277625089	-0.91768883402488	4.74776506966650
H	-2.16060410220921	-1.08421008149203	5.80256682472063
C	-0.70099289236054	-0.99389621409345	4.23215223626286
H	0.13937279977484	-1.21722540095063	4.87551855004015
C	-0.49530084711101	-0.78929216356087	2.87674459304944
H	0.51294019045388	-0.85561477116826	2.49390504955879
C	-4.06993702651092	-0.04539483603596	1.71787469618156
H	-4.12785758369828	1.03244377048110	1.55834582533716
H	-4.98553252757805	-0.34639308659579	2.22510255433660
H	-4.07658830964064	-0.52065877472447	0.73763885220821
Cl	3.03618267939344	-3.03722955141870	0.38967240869307
Cl	5.65564715511323	-1.31939615344748	0.22781380481118
Cl	5.45273520894098	1.76821359228099	-0.19420476846298
Cl	2.62467501859870	3.11040117722057	-0.42849829178716
O	0.57226259410179	-1.39163775875393	0.08173732665613
O	0.41326735872837	1.15647010989387	-0.26193513347212
Sb	-1.15436457213467	-0.12865398011970	-0.00914434850559

2 (chloroform phase)

C	1.71240521038717	-0.74562792518053	0.03003298418099
C	2.95943287941106	-1.33780638836476	0.15205086861912
C	4.12154331432932	-0.56363442217836	0.08183990287281
C	4.03361877122508	0.80774602542492	-0.10112795410665
C	2.77761301244376	1.41287370967029	-0.21329747772044
C	1.63188385017182	0.64588644573790	-0.15189419933127
C	-2.35118555903859	1.55629905899106	-0.58824014143189
C	-2.35034911732734	2.78424483959959	0.08942886052524
C	-3.17587001000476	3.79714986579203	-0.39842499173677
H	-3.19446935861655	4.74918185048527	0.11853705282996

C	-3.97324931063036	3.61608291059707	-1.51891342948021
H	-4.60016106358140	4.42571057022580	-1.86943877809147
C	-3.96936354444718	2.39797379592250	-2.17789608594995
H	-4.58958951176921	2.23883557190623	-3.04995684931822
C	-3.15931204963442	1.37433505196710	-1.70660095101593
H	-3.17552906623736	0.42445925164723	-2.22691839245879
C	-1.51951342549710	3.06982569776353	1.30713218772600
H	-1.50062648674186	2.23727056698097	2.01016050377516
H	-1.91193935351044	3.93417421298115	1.84096263013347
H	-0.48616316994595	3.29641141288660	1.04264461882457
C	-1.86031134253121	-1.62226414478753	-1.34567327216615
C	-1.39457683005752	-1.68627951899424	-2.66304286093852
C	-1.88405681137955	-2.70934320233282	-3.47110631764982
H	-1.54436779691724	-2.77543984799680	-4.49748941152235
C	-2.79211622319607	-3.64157678165156	-2.98818360393687
H	-3.15329992020388	-4.42535860967882	-3.64109290525008
C	-3.23046454286904	-3.57174650855310	-1.67579257023257
H	-3.93213008908890	-4.29917216371900	-1.29006264939696
C	-2.76060650167658	-2.55798561217634	-0.85104577654090
H	-3.09977545443772	-2.51194052569215	0.17703754430151
C	-0.39979257113625	-0.70663998529614	-3.20999480268279
H	-0.72570326993836	0.32711419687600	-3.07415429593074
H	-0.24754305273752	-0.85818677759426	-4.27730602963970
H	0.57373124705230	-0.81290003339029	-2.72695582767075
C	-1.56957290362968	-0.49967449985226	2.03770086679429
C	-2.87167763157700	-0.40354182644986	2.54571294279783
C	-3.05080939428420	-0.62790248409712	3.91077138582667
H	-4.05187545998829	-0.57151328095976	4.32044607569775
C	-1.98529277625089	-0.91768883402488	4.74776506966650
H	-2.16060410220921	-1.08421008149203	5.80256682472063
C	-0.70099289236054	-0.99389621409345	4.23215223626286
H	0.13937279977484	-1.21722540095063	4.87551855004015
C	-0.49530084711101	-0.78929216356087	2.87674459304944
H	0.51294019045388	-0.85561477116826	2.49390504955879
C	-4.06993702651092	-0.04539483603596	1.71787469618156
H	-4.12785758369828	1.03244377048110	1.55834582533716
H	-4.98553252757805	-0.34639308659579	2.22510255433660
H	-4.07658830964064	-0.52065877472447	0.73763885220821
Cl	3.03618267939344	-3.03722955141870	0.38967240869307
Cl	5.65564715511323	-1.31939615344748	0.22781380481118
Cl	5.45273520894098	1.76821359228099	-0.19420476846298
Cl	2.62467501859870	3.11040117722057	-0.42849829178716
O	0.57226259410179	-1.39163775875393	0.08173732665613
O	0.41326735872837	1.15647010989387	-0.26193513347212
Sb	-1.15436457213467	-0.12865398011970	-0.00914434850559

[2t_O-F]$^-$ (uuu, gas phase)

Sb	1.21101870400084	0.07086793611668	-0.50251996796477
Cl	-5.02640186660658	-0.11809229668067	2.28145737495617
Cl	-5.62977574615690	0.10053950252519	-0.77751552686904
Cl	-3.25297365489133	0.41166299393651	-2.80379501122789
Cl	-2.05100515976900	-0.03163706617225	3.27708033139943
F	2.21676074521057	-0.10026280853584	-2.16993580958855
O	-0.11161477779413	0.34196320511894	1.10000938534565
O	-0.62092808115747	0.55536885083987	-1.48220708457745
C	1.63043832707266	2.20506118937554	-0.53744107092045
C	-1.36735934197589	0.27812572008960	0.71453514541749
C	2.89596769267800	2.74756258855382	-0.81065512624285
C	-1.63710593274901	0.38523080544056	-0.67686346015836
C	0.57914223001885	3.06796711777064	-0.23979605970713
C	-4.00697231826353	0.15788239566835	-0.20507183673990
C	-2.41805447817858	0.10897382249176	1.60145878621449
C	-2.95352671543627	0.30630668615287	-1.11214608101154
C	0.75013173727393	4.44378703427790	-0.20553340253929

C	2.79693772161708	-0.38217236122035	0.91930564672846
C	4.10764936991778	1.92876345942194	-1.16177924194554
C	-3.74071509118264	0.06020513296748	1.15027217912371
C	1.99590266384561	4.98189927390985	-0.47312197902309
C	3.04904508783182	4.13411393994027	-0.76968895363168
C	0.51635663209644	-1.99118781486330	-0.63131935241531
C	2.82843153638154	0.42316500195144	2.05507878640437
C	0.42620540106574	-2.62745896608012	0.60448718940827
C	4.73411031056319	-1.53519123089366	1.75536906017282
C	3.76572399362203	-1.38438546922610	0.76314496408372
C	0.14694337426014	-2.68985672184153	-1.78918097595078
C	4.75534727025552	-0.72967451047391	2.88259768060396
C	3.79604973186051	0.25692589450445	3.03548065098582
C	-0.26591511222776	-4.01666809066363	-1.65540645538647
C	0.00590012279342	-3.94446565186787	0.71851079504059
C	3.81048266045509	-2.30658913917440	-0.42355911388628
C	-0.33605867222682	-4.64635869829210	-0.42369464666169
C	0.17063612002751	-2.09509100419076	-3.16882979035166
H	-0.40721918159318	2.68406582411530	-0.02617823576075
H	-0.09096509676216	5.08420242788381	0.02787389726279
H	4.31224348165329	1.15820424951398	-0.41954314813022
H	4.99059627176365	2.56542047587643	-1.22229032858360
H	3.98396276003259	1.42876150669577	-2.11921459530879
H	2.15110337902404	6.05355045145140	-0.45253095461621
H	4.02577690185214	4.55495514708689	-0.98078670175091
H	2.08884256109929	1.20389859785772	2.18415433365401
H	5.48938599681129	-2.30441238107963	1.63692218699861
H	5.52114117678962	-0.87343589648245	3.63492546945963
H	3.79679665895196	0.89547883575643	3.90973710674949
H	-0.55012072976665	-4.56500018004846	-2.54667822270683
H	-0.05829007020694	-4.40957805516943	1.69408300540658
H	3.00383785956751	-3.03979683600702	-0.38486764114390
H	4.75320608641252	-2.85423362338675	-0.45012361959483
H	3.70617748887654	-1.77090895815718	-1.36435207653416
H	-0.66672907882118	-5.67585478218705	-0.35965998228420
H	1.18272399113905	-1.84325467006893	-3.47876924995079
H	-0.24000674491122	-2.79959695722717	-3.89274915033224
H	-0.41676149038534	-1.18026534015853	-3.21865970077697
H	0.68922238424073	-2.09328457714231	1.50964074885810

$[2t_O\text{-F}]^-$ (uuu, water phase)

Sb	1.23826918062010	0.11949003425006	-0.47387044257989
Cl	-4.97800785645391	-0.60080586692707	2.20257126372310
Cl	-5.56969313345916	-0.29461806338902	-0.84878020871230
Cl	-3.22211699682856	0.32936726227559	-2.82427077518267
Cl	-2.04028297830746	-0.25790463518290	3.24483140012311
F	2.15385814755457	-0.04774726617456	-2.19750887930018
O	-0.12090796617907	0.40811574723218	1.11518242763115
O	-0.61784143478260	0.66094569998345	-1.46092013336826
C	1.66586069837310	2.24738929257005	-0.52856410705121
C	-1.36717744752603	0.25198484735666	0.71147841316661
C	2.93530121439014	2.76285416968686	-0.83968152833371
C	-1.63041306171261	0.38570658248943	-0.67712709863425
C	0.63894004872127	3.13176660979956	-0.19934943033970
H	-0.34853426071157	2.77020432549526	0.05033481896752
C	-3.97105454648441	-0.09766106584911	-0.25022861801524
C	-2.40323181691535	-0.06694962172450	1.57409619413489
C	-2.92832412455229	0.19403020464988	-1.13376884371725
C	0.83847907546165	4.50463596741300	-0.17366517597661
H	0.02038133296853	5.16355312018224	0.08695003355481
C	2.81663953153331	-0.34208715364697	0.93874934938736
C	4.12500168393329	1.92958105782509	-1.23002874680277
H	4.24501872086082	1.04228412180748	-0.61160057118869
H	5.03947273670086	2.51423477171906	-1.13750816591526

H	4.05725429355414	1.59713435644711	-2.26514805255819
C	-3.71008854831496	-0.22955388261655	1.10363468603515
C	2.08804524825812	5.01691664137389	-0.48173235000947
H	2.26528280771829	6.08459897245103	-0.46682456460498
C	3.11607740122602	4.14826950183403	-0.80743659184691
H	4.09419448744250	4.55042393833487	-1.04457192754671
C	0.47665233669512	-1.91669543503296	-0.61825305558992
C	2.88539267998900	0.49910327858773	2.04845875809924
H	2.18773563666100	1.32129289289237	2.15917254357043
C	0.37202453912884	-2.55153475495805	0.61921602889845
C	4.71567252165447	-1.54016014969937	1.79747421344712
H	5.44511827594969	-2.33546121189788	1.69674530786263
C	3.74833138927902	-1.38220663352093	0.80341777540497
C	0.07913123070355	-2.60392096650618	-1.77551898134611
C	4.77030153116863	-0.70436466658466	2.90237789632249
H	5.53395382371172	-0.85438498977967	3.65467517859730
C	3.85032378758742	0.32448110474766	3.03085235064266
H	3.88327658668891	0.98987254464897	3.88377743318118
C	-0.40090044296016	-3.90968280129192	-1.63408681110205
H	-0.70903946516063	-4.44919290539067	-2.52217193151172
C	-0.10844896259790	-3.84747598773437	0.73951089244085
H	-0.17824135470312	-4.31142693417538	1.71494803309871
C	3.76527472136967	-2.33510994642018	-0.35822975371050
H	2.97958713700255	-3.08715687962115	-0.27013800261167
H	4.71566790700932	-2.86584258634027	-0.40461834688854
H	3.62357986293066	-1.83377581050688	-1.31365449256276
C	-0.49745049792970	-4.53225860567939	-0.40009868826819
H	-0.87640953039543	-5.54401006457323	-0.33279250426982
C	0.15297476873624	-2.04793163554317	-3.17049215280746
H	1.18338980207574	-1.92054435073660	-3.49807534182796
H	-0.32875936637169	-2.72838392019361	-3.87166577657716
H	-0.33981090572774	-1.08256616028643	-3.26173374732650
H	0.67596864041589	-2.04062611406986	1.52532096979495

[2t_O-F]$^-$ (uuu, chloroform phase)

Sb	1.23280799343739	0.07906292850007	-0.47783343344905
Cl	-5.00417796716483	-0.32225975847196	2.26952073864294
Cl	-5.61146852247100	-0.01078944423022	-0.78216249420372
Cl	-3.24325912555534	0.42656194166196	-2.79312516549744
Cl	-2.03564378882799	-0.15900124757394	3.27542737170700
F	2.19213225209445	-0.08114108439648	-2.17776063801241
O	-0.10705173362248	0.34899559165827	1.11598462256583
O	-0.61371558611774	0.59010680843541	-1.46099045485470
C	1.64492169968520	2.21443666253963	-0.53401291166315
C	-1.35914985708962	0.25592585534788	0.72550519983496
C	2.90529910345828	2.75195242967519	-0.84331172097391
C	-1.62811467124134	0.38528079475858	-0.66473215063108
C	0.60079255206680	3.08344275203133	-0.22008432086100
H	-0.38045612018983	2.70598857379832	0.02846510150855
C	-3.99313252877182	0.07549148316034	-0.20395896117993
C	-2.40526820410424	0.02302449454463	1.60335770933147
C	-2.94223071572229	0.28150078304297	-1.10359487097641
C	0.77308538823705	4.46004116305061	-0.20946303833428
H	-0.06024128333685	5.10521905825630	0.03829714567524
C	2.81385827690403	-0.36979581726748	0.94447123190601
C	4.11235032422769	1.93457694808429	-1.21355753079370
H	4.24708851900551	1.06636120742668	-0.57153707326687
H	5.01600134600451	2.53863341801107	-1.13383812738192
H	4.05108335498403	1.57302239186473	-2.23917460606846
C	-3.72590421491846	-0.05698525843060	1.14852969787549
C	2.01337793998287	4.99344884723691	-0.51790359220190
H	2.16926554441809	6.06493850839595	-0.51654432814452
C	3.05973096813420	4.14079099610729	-0.82660077913766
H	4.03143546684626	4.55879242289892	-1.06404172991022

C	0.50745275580219	-1.97115263017211	-0.63229302629602
C	2.84824894435175	0.45165957658699	2.07022430615739
H	2.12079336789988	1.24591355127940	2.18928785928514
C	0.40619341542421	-2.61814230180050	0.59859095614702
C	4.73726346375144	-1.53001516253271	1.80437911930134
H	5.48787031525323	-2.30527228793720	1.69934424394149
C	3.77513498186429	-1.38242792473167	0.80367651891965
C	0.11674235503102	-2.65075440338070	-1.79620248762761
C	4.75920053537657	-0.71100547001878	2.92281175102615
H	5.51964959187651	-0.85238546821886	3.68057395599903
C	3.80888643499046	0.28846928882010	3.05876838564304
H	3.81282778195143	0.93929899213399	3.92387529130002
C	-0.34196502560602	-3.96523658989355	-1.67018325559409
H	-0.64428568119768	-4.49890059834793	-2.56420157707615
C	-0.05556508339696	-3.92247666248500	0.70465548179553
H	-0.12496096121969	-4.39568048870212	1.67601692611189
C	3.82371608255047	-2.31946652218698	-0.37048351114661
H	3.03805874602617	-3.07424313450733	-0.31039699332239
H	4.77712311747248	-2.84688587197168	-0.40317053664808
H	3.70000461731723	-1.80507567192247	-1.32132654432727
C	-0.42910317738140	-4.60243601298130	-0.44271639888310
H	-0.79303967597902	-5.62077626148839	-0.38603753077330
C	0.17368959738817	-2.06488702355972	-3.17934908130198
H	1.20048487567365	-1.95300168718896	-3.52553263232689
H	-0.33987550344217	-2.71577795516274	-3.88699338606021
H	-0.29541565789978	-1.08487899256241	-3.22843472047572
H	0.69315246576874	-2.10588975718397	1.50944616472666

[2*t*$_O$-F]$^-$ (uud, gas phase)

Sb	6.28629399456686	14.09629565033575	9.11364802037720
Cl	7.03048302188036	10.77758733353533	15.03544455124334
Cl	9.61937006389154	10.00413298040793	13.46592629293366
Cl	9.99601877895154	11.00373876617561	10.51570772623024
Cl	4.84375531627088	12.53742776804248	13.63216809473766
F	7.30929982460444	14.63697608028685	7.53902614908172
O	5.51345307651151	13.21747193538907	10.84957986313787
O	7.68628705084059	12.53789872087021	9.51253572109993
C	5.21401405881577	12.62399240645058	7.91097216187389
C	6.41911132434412	12.49349550798594	11.47442097371757
C	4.62192798259547	12.90396483858029	6.66909903332524
C	7.59037359343445	12.13863677037254	10.75206582807735
C	5.08409116727985	11.34178552886569	8.43921216885787
H	5.52982687134482	11.08818217348790	9.38924338993257
C	8.40071073697762	10.96012661327085	12.71365850364379
C	6.27289473230157	12.07908826321761	12.78801863760118
C	8.56963115149305	11.39099703559390	11.39468550127435
C	4.38753990490318	10.34248701934304	7.77605118925676
H	4.31134878600982	9.35692466428586	8.21762869201584
C	4.64885589939648	15.52230251890567	9.01757965153487
C	4.68267096722872	14.24497547941974	5.99018645918166
H	4.09904971738945	14.99233079575365	6.52877668683844
H	4.27566134097309	14.17875012850317	4.98083225951580
H	5.70149832183501	14.61561900716600	5.91898762221271
C	7.25403675787962	11.30136782329139	13.41076885756224
C	3.80165158631490	10.61660992294208	6.55366690802575
H	3.25448616408526	9.85032262690952	6.01847556737124
C	3.92511286503391	11.88610060408566	6.01588497362478
H	3.46988664155173	12.10165083603717	5.05560502724495
C	7.65523752549910	15.29690094309684	10.31965329147443
C	3.40305961547440	15.02293339320816	9.38678178242029
H	3.30428365101237	13.99084081575948	9.70105151816486
C	7.29964732445834	16.06104289508173	11.44053081771341
C	3.60793099670715	17.64352042198081	8.57927358915780
H	3.68596941274624	18.67666562397523	8.25930630695387

C	4.75923097303520	16.85782571155373	8.60811243755627
C	8.99564514331077	15.23363264995062	9.94085816954555
H	9.28848198231639	14.63508970298852	9.09009057719791
C	2.37185717701435	17.13846299781380	8.95167883671960
H	1.49617939044488	17.77496536456251	8.91996159094123
C	2.26740321199051	15.81990760029335	9.36083313781677
H	1.31048067374268	15.40878275744243	9.65663304610513
C	9.98200326305010	15.91156974384455	10.63935869241366
H	11.01546425432825	15.83398792289636	10.32589380541784
C	8.30870294575696	16.73658898640894	12.13122168250377
H	8.03849237397420	17.32203090952327	13.00291701141039
C	6.06695661448496	17.47803420827130	8.20316763426517
H	6.79468806331111	17.44854759695255	9.01457203852508
H	5.92871911797438	18.52180675657721	7.92053856388763
H	6.51981670311133	16.95663987741040	7.36181086129643
C	9.63525060668421	16.67024115677590	11.74427790699316
H	10.39205252079244	17.20016925373444	12.30955877742608
C	5.89067021102214	16.22042956140095	11.94414702078035
H	5.29210404891514	16.84405935272607	11.27935297669725
H	5.89273066845254	16.69817292313783	12.92417697191142
H	5.37202983168397	15.27001907312081	12.04156644317374

[2t_O-F]$^-$ (udd, gas phase)

Sb	1.16934948758894	0.05934134044065	-0.58791586659630
Cl	-5.02034349149774	-0.33318740309623	2.25498779629315
Cl	-5.66770113679246	0.18304981321635	-0.75944399355513
Cl	-3.32068405189073	0.68085387568520	-2.78236546899358
Cl	-2.03036303649279	-0.32035715794624	3.21453878080145
F	2.03029247007149	-0.05541816834543	-2.33901608556405
O	-0.12252698741909	0.24318124146588	1.04738356364051
O	-0.66562204010593	0.66125775216570	-1.49517223697301
C	1.64581501927868	2.18359565083428	-0.50083928610003
C	-1.38665803921847	0.21180666241179	0.67726879683299
C	2.92124271050818	2.71056073833738	-0.75953451060272
C	-1.67239474998850	0.43781850801301	-0.69583654882676
C	0.63050045691784	3.04948456535979	-0.10122231184803
H	-0.36242294970979	2.67708688538713	0.10287248198193
C	-4.03655303159883	0.18491528440602	-0.20840602427005
C	-2.42246391347338	-0.03504867528499	1.56174939283488
C	-2.99695513338049	0.41403515615273	-1.11459398641630
C	0.84825625331264	4.41013441286054	0.05418594547212
H	0.03490353202921	5.05286828749886	0.36650255358893
C	2.89270522702201	-0.59764969567236	0.56481773179303
C	4.10942388769969	1.88742652440076	-1.17749617045298
H	4.51143131608280	1.31309720677134	-0.34185044249782
H	4.90889317440155	2.53438317754146	-1.54047211499196
H	3.86110176288330	1.18153548506589	-1.96402521689987
C	-3.75193322477263	-0.04116722163201	1.12798439252541
C	2.10468232757903	4.93237448801975	-0.19670854431918
H	2.29574655817078	5.99240416824770	-0.08290892249680
C	3.11986418589853	4.08340662285072	-0.60136376509065
H	4.10362789596357	4.49216974640680	-0.80418254630714
C	0.43919000089934	-1.96513539743701	-0.96558948006974
C	3.28662185868779	-0.11558657518671	1.82017510882782
C	0.33059397502802	-2.98276184183296	-0.00758859209392
C	4.79883962813892	-2.09781855776302	0.53507588100594
H	5.37414294410535	-2.86392736068063	0.03061499334799
C	3.65600137862294	-1.58153984013588	-0.05793367835932
C	0.03094617186675	-2.22025666911970	-2.27453342645051
H	0.09052577077687	-1.44010552178555	-3.01996312479620
C	5.19135199992571	-1.62454790057402	1.77585825761186
H	6.07937987146409	-2.01452820547691	2.25792348831463
C	4.43735864103504	-0.64585984224245	2.40349775975040
H	4.74646766100503	-0.27925248250439	3.37611463254181

C	-0.46417201960353	-3.45755813924475	-2.65400883509395
H	-0.77614496087064	-3.62283501613237	-3.67767075765787
C	-0.17405057660055	-4.22196652683200	-0.40955028881821
H	-0.26722730711513	-5.00929513518305	0.33027184159946
C	-0.56588857524168	-4.46798848246234	-1.71302855316405
H	-0.95790612002245	-5.43982797696116	-1.98706986925254
C	0.73851454633560	-2.84113772849476	1.43386781274654
H	1.82183318873456	-2.89802908469996	1.54757334253973
H	0.30441624386301	-3.64519524884733	2.02889378029184
H	0.41578539306629	-1.90092638086675	1.87253345901706
C	2.52619377800024	0.94087579810498	2.57139569285828
H	2.44231497707357	1.86513341452490	1.99998726542665
H	1.51081471123856	0.62156338047181	2.80145398876889
H	3.02607659737897	1.17862797411491	3.51071076768275
H	3.35388316313983	-1.96676944431532	-1.02500673953735

[2t_{O} -F]$^{-}$ (ddd, gas phase)

Sb	1.22234080262893	0.04705319688662	-0.60298866520078
Cl	-4.92773969724852	-0.32067567765264	2.27841099106063
Cl	-5.58014158262896	0.39959041879974	-0.69200083109573
Cl	-3.23621685968390	1.00863626734621	-2.68729215512697
Cl	-1.93303881113747	-0.42077530727214	3.21948383180900
F	2.07057402681343	-0.05297845560210	-2.35927546465404
O	-0.02462129845174	0.20864920604061	1.07097592769205
O	-0.57245831812808	0.84071056362116	-1.42594947118747
C	2.02458430605353	2.07343273845740	-0.45557887110250
C	-1.29222193488130	0.23694260382465	0.71088583827195
C	3.36898146680797	2.13116504992665	-0.82253420873819
C	-1.58104151008533	0.57175711450121	-0.63813499498977
C	1.40313638947421	3.24296510694851	0.00707226815146
C	-3.94668917185023	0.33761496526634	-0.15202060465066
C	-2.32806960315140	-0.03466186517497	1.58779610110051
C	-2.90739099207651	0.60815447627027	-1.04729093695574
C	2.16297565166909	4.41224833149958	0.09017554090185
H	1.68467366457367	5.31537572840459	0.45243867492008
C	2.92171904969832	-0.80226376383344	0.45824214114243
C	-3.65962134997866	0.01765689992628	1.16437096793113
C	3.49859047215166	4.45327060044125	-0.26931506984427
H	4.05497204630717	5.37889852382222	-0.18607630266432
C	4.10797884671891	3.30070887836839	-0.73197076065169
H	5.15119172427307	3.30523671507142	-1.02192049493641
C	0.31398627182188	-1.88294394649782	-1.08366747360045
C	3.38189642980383	-0.41587663257866	1.72420901108280
C	0.12936129485440	-2.94683187953929	-0.18915889395105
C	4.67605996041382	-2.47165606557858	0.32288402903516
H	5.16719344876795	-3.26487077128446	-0.22665581193998
C	3.57897744461384	-1.82189074538839	-0.22501215557728
C	-0.14675623832080	-2.01219371786835	-2.39366898757168
H	-0.02651989099684	-1.19512451879930	-3.09073018272742
C	5.12998857147525	-2.09662232828772	1.57589629708321
H	5.98251716462398	-2.59290382916456	2.02313640428021
C	4.48547494551546	-1.07838566726299	2.26025832601942
H	4.84509568252042	-0.78739387077892	3.24102333183264
C	-0.77557950414155	-3.16588826000882	-2.83335063596707
H	-1.12922589249049	-3.23138093145243	-3.85471366254156
C	-0.50701445401656	-4.10118491417412	-0.65187095524841
H	-0.65909341770812	-4.92257050857020	0.03949362630577
C	-0.95746338732663	-4.21994322091116	-1.95447299107619
H	-1.45565836777197	-5.12650707684011	-2.27562614647061
C	0.58755002632913	-2.93945782322012	1.24427413372633
H	1.66962497633157	-3.05411240720202	1.31744976722632
H	0.13273771536686	-3.76633894202538	1.79040965187947
H	0.32506403668258	-2.02200331508482	1.76398752694824
H	3.87061048584583	1.24189407206292	-1.18090313668628

C	-0.03239325661911	3.32601051553135	0.43873756748546
H	-0.70837769164277	3.08152488681757	-0.37748721624789
H	-0.24647214018339	2.64816603870485	1.26224969783969
H	-0.27145260017589	4.33530622675255	0.77445022025813
H	3.22417755050205	-2.13106607753294	-1.20128458985052
C	2.73511766722074	0.67129925609657	2.53541029923119
H	2.73933928982826	1.62381866775731	2.00540561543479
H	1.69639456197853	0.44024724927343	2.76714230978306
H	3.26490797902978	0.81667955116680	3.47723904282197

[2t_O-F]$^-$ (ddd, gas phase)

Sb	-1.05334759722656	0.04470121959404	-0.41379367229642
Cl	2.85970263565863	3.19166767825880	0.04655613283158
Cl	2.90200881721757	-3.02340701798205	0.04942309812222
Cl	5.57574246917781	1.66626495853198	0.45224811901866
Cl	5.59956098876579	-1.45903125276720	0.45473409197654
F	-0.68439843359279	0.04519757644365	-2.33403220569116
O	0.58750363078340	-1.24853011044180	-0.29616340021557
O	0.56072754518042	1.37962750443017	-0.28360971197433
C	-1.10480996228237	0.03802244636643	1.78065784094292
C	-2.37603986383084	1.77141112446079	-0.62526046770721
C	1.71483789566660	0.78583907932984	-0.11979861404813
C	1.72949458559927	-0.63208222530116	-0.12352158752924
C	-2.43380775137534	-1.60247761728459	-0.75300612517074
C	-3.40492502258255	1.87340565603812	0.31025606400516
C	-3.62581461458354	-1.53275147047175	-0.03641985266311
C	-4.35178472745604	2.88579498343482	0.27400063020904
C	2.90629521900371	1.47103696298604	0.05580414964013
C	-4.40801473103707	-3.58378768977440	-0.97278549858105
C	-4.61347237018394	-2.50070467454915	-0.13801143511526
C	-3.22534006402451	-3.67110702198590	-1.68766519324588
C	-1.03321162079208	1.29677645866578	2.37659827399429
C	2.92830244745642	-1.30130393316707	0.05502458696068
C	-1.00470847363951	1.45814998755378	3.75320427014508
C	-4.27083944817003	3.84546871430553	-0.71738593734037
C	-3.23579907095426	3.78099648381785	-1.63407072069331
C	-2.27422450327507	2.76823343547521	-1.61162260807826
C	-2.22595976370580	-2.69909127517130	-1.60562989129131
C	4.12492133960666	-0.59868772324513	0.23322570169711
C	-1.08124360966839	-0.91864719470862	3.98809016827344
C	-1.10342948470519	-1.09987356406631	2.60329519467754
C	4.11328430318778	0.78486376714532	0.23247719461192
C	-1.03800432530829	0.33835597205731	4.56604164346534
C	-1.18146881548079	2.84200119462889	-2.64072720352253
C	-0.99133545120289	-2.90481601342649	-2.43686944492244
C	-1.10980713648324	-2.51429046620189	2.09055911941446
H	-3.46594535251204	1.16569169162072	1.12626611234055
H	-3.79920794926129	-0.71368050372670	0.64728473216214
H	-5.13311868842089	2.92327740397896	1.02252542326385
H	-5.52621794139901	-2.40689724548039	0.43674811135931
H	-3.06469964887207	-4.52346881889652	-2.33803716923533
H	-0.99515998127087	2.18402479887402	1.75705541134052
H	-0.94646833831032	2.45110657551155	4.18075779173577
H	-3.15785191792923	4.54936185802828	-2.39477478710121
H	-1.08344095619917	-1.79521371176952	4.62638779405155
H	-0.19702764571833	2.75073086831790	-2.18784570649415
H	-1.26907795640234	2.04833516518455	-3.37837269940842
H	-1.22715565607208	3.79866421378704	-3.16209064979978
H	-0.89604760187800	-2.13351112040509	-3.19745935999598
H	-0.08578833346338	-2.86755369715149	-1.83609134212766
H	-1.03050805498813	-3.87402829136725	-2.93491006166109
H	-2.07279347155561	-2.78922144532353	1.66076018904552
H	-0.35667656883770	-2.67075264863750	1.32202192432327
H	-0.90328033511321	-3.21062238339671	2.90399802766874

H	-1.01555587197637	0.43843170281562	5.64434998910110
H	-5.15830325754473	-4.35916494294522	-1.06757829782528
H	-4.99479883801812	4.64927680800171	-0.77030250264322

[2t_O-F]$^-$ (ddd, gas phase)

Sb	-1.05334759722656	0.04470121959404	-0.41379367229642
Cl	2.85970263565863	3.19166767825880	0.04655613283158
Cl	2.90200881721757	-3.02340701798205	0.04942309812222
Cl	5.57574246917781	1.66626495853198	0.45224811901866
Cl	5.59956098876579	-1.45903125276720	0.45473409197654
F	-0.68439843359279	0.04519757644365	-2.33403220569116
O	0.58750363078340	-1.24853011044180	-0.29616340021557
O	0.56072754518042	1.37962750443017	-0.28360971197433
C	-1.10480996228237	0.03802244636643	1.78065784094292
C	-2.37603986383084	1.77141112446079	-0.62526046770721
C	1.71483789566660	0.78583907932984	-0.11979861404813
C	1.72949458559927	-0.63208222530116	-0.12352158752924
C	-2.43380775137534	-1.60247761728459	-0.75300612517074
C	-3.40492502258255	1.87340565603812	0.31025606400516
C	-3.62581461458354	-1.53275147047175	-0.03641985266311
C	-4.35178472745604	2.88579498343482	0.27400063020904
C	2.90629521900371	1.47103696298604	0.05580414964013
C	-4.40801473103707	-3.58378768977440	-0.97278549858105
C	-4.61347237018394	-2.50070467454915	-0.13801143511526
C	-3.22534006402451	-3.67110702198590	-1.68766519324588
C	-1.03321162079208	1.29677645866578	2.37659827399429
C	2.92830244745642	-1.30130393316707	0.05502458696068
C	-1.00470847363951	1.45814998755378	3.75320427014508
C	-4.27083944817003	3.84546871430553	-0.71738593734037
C	-3.23579907095426	3.78099648381785	-1.63407072069331
C	-2.27422450327507	2.76823343547521	-1.61162260807826
C	-2.22595976370580	-2.69909127517130	-1.60562989129131
C	4.12492133960666	-0.59868772324513	0.23322570169711
C	-1.08124360966839	-0.91864719470862	3.98809016827344
C	-1.10342948470519	-1.09987356406631	2.60329519467754
C	4.11328430318778	0.78486376714532	0.23247719461192
C	-1.03800432530829	0.33835597205731	4.56604164346534
C	-1.18146881548079	2.84200119462889	-2.64072720352253
C	-0.99133545120289	-2.90481601342649	-2.43686944492244
C	-1.10980713648324	-2.51429046620189	2.09055911941446
H	-3.46594535251204	1.16569169162072	1.12626611234055
H	-3.79920794926129	-0.71368050372670	0.64728473216214
H	-5.13311868842089	2.92327740397896	1.02252542326385
H	-5.52621794139901	-2.40689724548039	0.43674811135931
H	-3.06469964887207	-4.52346881889652	-2.33803716923533
H	-0.99515998127087	2.18402479887402	1.75705541134052
H	-0.94646833831032	2.45110657551155	4.18075779173577
H	-3.15785191792923	4.54936185802828	-2.39477478710121
H	-1.08344095619917	-1.79521371176952	4.62638779405155
H	-0.19702764571833	2.75073086831790	-2.18784570649415
H	-1.26907795640234	2.04833516518455	-3.37837269940842
H	-1.22715565607208	3.79866421378704	-3.16209064979978
H	-0.89604760187800	-2.13351112040509	-3.19745935999598
H	-0.08578833346338	-2.86755369715149	-1.83609134212766
H	-1.03050805498813	-3.87402829136725	-2.93491006166109
H	-2.07279347155561	-2.78922144532353	1.66076018904552
H	-0.35667656883770	-2.67075264863750	1.32202192432327
H	-0.90328033511321	-3.21062238339671	2.90399802766874
H	-1.01555587197637	0.43843170281562	5.64434998910110
H	-5.15830325754473	-4.35916494294522	-1.06757829782528
H	-4.99479883801812	4.64927680800171	-0.77030250264322

[2t_C-F]$^-$ (gas phase)

Sb	-1.05334759722656	0.04470121959404	-0.41379367229642

Cl	2.85970263565863	3.19166767825880	0.04655613283158
Cl	2.90200881721757	-3.02340701798205	0.04942309812222
Cl	5.57574246917781	1.66626495853198	0.45224811901866
Cl	5.59956098876579	-1.45903125276720	0.45473409197654
F	-0.68439843359279	0.04519757644365	-2.33403220569116
O	0.58750363078340	-1.24853011044180	-0.29616340021557
O	0.56072754518042	1.37962750443017	-0.28360971197433
C	-1.10480996228237	0.03802244636643	1.78065784094292
C	-2.37603986383084	1.77141112446079	-0.62526046770721
C	1.71483789566660	0.78583907932984	-0.11979861404813
C	1.72949458559927	-0.63208222530116	-0.12352158752924
C	-2.43380775137534	-1.60247761728459	-0.75300612517074
C	-3.40492502258255	1.87340565603812	0.31025606400516
C	-3.62581461458354	-1.53275147047175	-0.03641985266311
C	-4.35178472745604	2.88579498343482	0.27400063020904
C	2.90629521900371	1.47103696298604	0.05580414964013
C	-4.40801473103707	-3.58378768977440	-0.97278549858105
C	-4.61347237018394	-2.50070467454915	-0.13801143511526
C	-3.22534006402451	-3.67110702198590	-1.68766519324588
C	-1.03321162079208	1.29677645866578	2.37659827399429
C	2.92830244745642	-1.30130393316707	0.05502458696068
C	-1.00470847363951	1.45814998755378	3.75320427014508
C	-4.27083944817003	3.84546871430553	-0.71738593734037
C	-3.23579907095426	3.78099648381785	-1.63407072069331
C	-2.27422450327507	2.76823343547521	-1.61162260807826
C	-2.22595976370580	-2.69909127517130	-1.60562989129131
C	4.12492133960666	-0.59868772324513	0.23322570169711
C	-1.08124360966839	-0.91864719470862	3.98809016827344
C	-1.10342948470519	-1.09987356406631	2.60329519467754
C	4.11328430318778	0.78486376714532	0.23247719461192
C	-1.03800432530829	0.33835597205731	4.56604164346534
C	-1.18146881548079	2.84200119462889	-2.64072720352253
C	-0.99133545120289	-2.90481601342649	-2.43686944492244
C	-1.10980713648324	-2.51429046620189	2.09055911941446
H	-3.46594535251204	1.16569169162072	1.12626611234055
H	-3.79920794926129	-0.71368050372670	0.64728473216214
H	-5.13311868842089	2.92327740397896	1.02252542326385
H	-5.52621794139901	-2.40689724548039	0.43674811135931
H	-3.06469964887207	-4.52346881889652	-2.33803716923533
H	-0.99515998127087	2.18402479887402	1.75705541134052
H	-0.94646833831032	2.45110657551155	4.18075779173577
H	-3.15785191792923	4.54936185802828	-2.39477478710121
H	-1.08344095619917	-1.79521371176952	4.62638779405155
H	-0.19702764571833	2.75073086831790	-2.18784570649415
H	-1.26907795640234	2.04833516518455	-3.37837269940842
H	-1.22715565607208	3.79866421378704	-3.16209064979978
H	-0.89604760187800	-2.13351112040509	-3.19745935999598
H	-0.08578833346338	-2.86755369715149	-1.83609134212766
H	-1.03050805498813	-3.87402829136725	-2.93491006166109
H	-2.07279347155561	-2.78922144532353	1.66076018904552
H	-0.35667656883770	-2.67075264863750	1.32202192432327
H	-0.90328033511321	-3.21062238339671	2.90399802766874
H	-1.01555587197637	0.43843170281562	5.64434998910110
H	-5.15830325754473	-4.35916494294522	-1.06757829782528
H	-4.99479883801812	4.64927680800171	-0.77030250264322

3 (gas phase)

C	1.86234180389887	0.82249893800364	0.12336430206658
C	2.96551429981258	1.64584374641100	0.02328375711495
C	4.23938323142193	1.08748320804735	-0.11655497172539
C	4.38737388875301	-0.29291311224769	-0.16515092120702
C	3.26703399689517	-1.12150578450088	-0.07456410243073
C	2.00521218953078	-0.57078883541074	0.07837982399729
C	-2.24778711313026	1.50151094363053	0.15756635410609

C	-3.59725935266736	1.17007937685568	0.27182243063274
H	-3.90636885527770	0.13817882145879	0.40287265064508
C	-4.57310617185294	2.15134114481011	0.21445791363545
H	-5.61607001511987	1.87004540571308	0.30052922048791
C	-4.23129402357091	3.49061382851247	0.04303647545678
C	-2.88334483493098	3.81277973976848	-0.06837516214611
H	-2.59159371641765	4.84732464677081	-0.20477069960368
C	-1.89810290231930	2.83675662315957	-0.01410871045147
H	-0.86188436529419	3.12998923729545	-0.10743647456638
C	-5.28809100480823	4.55381189171126	0.00411179552475
H	-6.18513927985100	4.21003116567375	-0.51037568068064
H	-4.93612781393846	5.45154700578977	-0.50227032817512
H	-5.58546383568324	4.84501869096697	1.01337938295757
C	-1.44696058364671	-1.12250598982302	1.88338918384488
C	-2.15619726369135	-0.43801178868657	2.86172318018693
H	-2.41230808904921	0.60641875869481	2.73524172174374
C	-2.54125119028672	-1.08983352454237	4.02468892331038
H	-3.08592307474059	-0.54085480435391	4.78306395592330
C	-2.23730625606920	-2.43000239768854	4.22983387680024
C	-1.52653202656439	-3.10450027433146	3.23807452834570
H	-1.27475037174559	-4.14906192527052	3.37775230822329
C	-1.12334266929574	-2.46325286401002	2.08070015668603
H	-0.54854029362031	-3.01194837690695	1.34779423379207
C	-2.63587381334083	-3.13405847128512	5.49216308326344
H	-1.76557332529526	-3.32272495717112	6.12297081795608
H	-3.09716343357675	-4.09924743562716	5.28240374223187
H	-3.34269538269470	-2.54668080030862	6.07609024219930
C	-1.32493373508400	-0.97235950542626	-1.73031674037434
C	-2.27822406564564	-0.36229673437156	-2.53736493197187
H	-2.76393582441706	0.55446489411328	-2.22967789070719
C	-2.61035219387563	-0.91819771987580	-3.76406057465639
H	-3.34894281810996	-0.42561545920927	-4.38466507474391
C	-2.00726543536970	-2.08864601482055	-4.20911194593787
C	-1.04780986423079	-2.68505969015489	-3.39350902570479
H	-0.55331854842167	-3.59060967060931	-3.72445375416564
C	-0.70075566446442	-2.13905912950558	-2.17028538353770
H	0.06887855666965	-2.61433382488551	-1.57906556921023
C	-2.37754895568948	-2.70441905501527	-5.52509211701377
H	-1.49326287500806	-3.00265169643104	-6.08821333441343
H	-2.95182658491967	-2.01762699761524	-6.14488202182739
H	-2.98403449830408	-3.60014965102559	-5.37903885485531
Cl	2.73471067898394	3.34417284877967	0.07217850373484
Cl	5.60748016820316	2.11262096851443	-0.23316520627035
Cl	5.94320806432409	-0.98868262269829	-0.34005900178855
Cl	3.40372106941203	-2.82933682297313	-0.15414291301584
O	0.61857530503424	1.29097777960495	0.24704228651721
O	0.90012307971674	-1.27876597636426	0.17067115750412
Sb	-0.83329421063649	-0.10300775113957	0.13892538229247

3 (water phase)

C	1.84442060808148	0.88419571073982	0.17116631534150
C	2.93440310468168	1.72951509475997	0.11850838427430
C	4.22803339659667	1.20436116900094	0.03013019485528
C	4.40950934681983	-0.16912280610988	-0.01479754467053
C	3.30481204342065	-1.02380040173984	0.02532153193659
C	2.01998796240073	-0.51022880591620	0.12347534758235
C	-2.27014249481868	1.52010342735313	0.12978498780488
C	-3.58813096025400	1.21530212366476	0.45867440142431
H	-3.86852394572970	0.21272112183581	0.76159622536596
C	-4.57395711624487	2.19077654430206	0.39728634643436
H	-5.59375148592880	1.93170353259014	0.65534591605866
C	-4.27132409952716	3.49138979251999	0.00530227822389
C	-2.95055777583729	3.78588454254711	-0.32913496330740
H	-2.69082943388030	4.78935028278663	-0.64546369931452

C	-1.95950389807403	2.81737887905405	-0.27209027706485
H	-0.95161797319160	3.09215189940793	-0.55063313424152
C	-5.32721007600680	4.55272650815454	-0.04360377723178
H	-6.32807697953277	4.12448275699215	-0.01158868330755
H	-5.24436954433791	5.15564231714434	-0.94807301764825
H	-5.23441186087606	5.23481327619896	0.80374730622886
C	-1.44228341584646	-1.13250225155903	1.85828028712983
C	-1.82260975349388	-0.42215438107102	2.98968542319959
H	-1.84079568733041	0.66098659851262	2.98798959457609
C	-2.18648380510105	-1.10479817662647	4.14299372306028
H	-2.47742057693376	-0.54232624549250	5.02170229099760
C	-2.18679940958370	-2.49576033970340	4.18380251534060
C	-1.80593059279889	-3.19326643612421	3.03623635049726
H	-1.80282963694645	-4.27670388875348	3.04462628729438
C	-1.42923093788988	-2.52489196087812	1.88379396372313
H	-1.14012174697100	-3.09777315585114	1.01148277299402
C	-2.58274974254019	-3.23707897737626	5.42355700146941
H	-1.74721867253790	-3.81905783514995	5.81572296131648
H	-3.39212805847807	-3.93953625325298	5.22103417656897
H	-2.91435910689420	-2.55880335209758	6.20806511300842
C	-1.32466302360457	-0.98262966714876	-1.73399229072033
C	-2.15748617962183	-0.30056675766477	-2.61336526881788
H	-2.55530736612869	0.67525249439786	-2.36490215749368
C	-2.48675492157007	-0.86520648593183	-3.83840275100957
H	-3.12958521664762	-0.31810055793910	-4.51717943826586
C	-2.00352642184113	-2.11709769202496	-4.20548959199988
C	-1.17260425803763	-2.79195736720962	-3.31021512458326
H	-0.78320679130913	-3.76820143083144	-3.57362199200611
C	-0.82859170645160	-2.23625740453861	-2.08965155381743
H	-0.17481924009322	-2.78878512485807	-1.42974491082742
C	-2.36817840235836	-2.73769738003666	-5.51864698607084
H	-1.48592197716946	-3.10973842131315	-6.04007495375768
H	-2.87348788085193	-2.02805616845136	-6.17172776161555
H	-3.03539639057360	-3.58949541732833	-5.37473784597002
Cl	2.66890405520662	3.42573710288707	0.16575576406086
Cl	5.57738723727937	2.26403416309947	-0.02283001491275
Cl	5.99050451736674	-0.82807088577013	-0.11911531087436
Cl	3.49661794753967	-2.72957891384410	-0.04447928128493
O	0.59321045728011	1.32457330367131	0.25984330273438
O	0.93491356971452	-1.24094961311422	0.17149812682569
Sb	-0.86780568254309	-0.08808808591338	0.12395344048598

3 (chloroform phase)

C	1.86142073171571	0.91778444845905	0.25797530355028
C	2.94945208126394	1.76165447219896	0.35412000552919
C	4.24661542845331	1.23841076978921	0.32694231965519
C	4.43444201877862	-0.12989573971537	0.19641483070769
C	3.33177861793624	-0.98222963100330	0.09067438442465
C	2.04412739400089	-0.46928181226081	0.12354659411450
C	-2.23461246999491	1.54486253803181	0.06275079562646
C	-3.55543143513048	1.24963608693464	0.38850992627213
H	-3.84543895254732	0.24572844332049	0.67841508131155
C	-4.53120394316973	2.23618589817248	0.34135160165859
H	-5.55347169561804	1.98437520515368	0.59760833941861
C	-4.21512060098808	3.53854449407694	-0.03338531624480
C	-2.89097095947447	3.82388836134074	-0.36241387682006
H	-2.61972976881377	4.82978421390725	-0.66170967368757
C	-1.90999812598799	2.84489221172844	-0.31851863097689
H	-0.89654349521730	3.11157311286631	-0.58477505715538
C	-5.25961520064555	4.61256298247921	-0.06906282153381
H	-6.26562833134644	4.19770957531921	-0.01804274667635
H	-5.18731751785029	5.20957799402049	-0.97881937858435
H	-5.14440586215924	5.29881492958923	0.77247373744761
C	-1.40599734027416	-1.12055225877863	1.77250711323749

C	-1.78764606245222	-0.41903729515680	2.90896468671646
H	-1.81177427203607	0.66417760705765	2.91306255026903
C	-2.14478020177082	-1.10903589954788	4.05999459178141
H	-2.43629640791524	-0.55191078700647	4.94212392871541
C	-2.13604505108393	-2.49993134483584	4.09479137329467
C	-1.75108414157610	-3.18899696016787	2.94359464012504
H	-1.73724437631310	-4.27260632959624	2.94778344626764
C	-1.38112937329810	-2.51269728618732	1.79378974564601
H	-1.08309755113257	-3.08078506707894	0.92155195435208
C	-2.52502140687632	-3.25054478496534	5.33212146097819
H	-1.68691841569522	-3.83356094179512	5.71795992412555
H	-3.33537349578895	-3.95263000734631	5.13019332006712
H	-2.85441778093162	-2.57891703416548	6.12367557127360
C	-1.34316131269657	-0.98558155135623	-1.79444347400712
C	-2.33747245475840	-0.40945072434446	-2.57652499882588
H	-2.81275493050180	0.51769191281730	-2.28283527152888
C	-2.73262696848526	-1.01773732048672	-3.76059545850012
H	-3.50468255735760	-0.55244271193890	-4.36141696572757
C	-2.15487593008308	-2.21031530947353	-4.18344607895555
C	-1.15605753407875	-2.77547289787269	-3.38980486516971
H	-0.68664782723006	-3.70175320948449	-3.70003048727191
C	-0.74683301833714	-2.17595196809348	-2.21095271660637
H	0.04036859746370	-2.63888361356776	-1.63312435774989
C	-2.58347027840367	-2.87847147375050	-5.45427597745252
H	-1.73886386662223	-3.01931128949383	-6.13045216724052
H	-3.33920118346910	-2.29718459286205	-5.98052669059755
H	-3.00141988335593	-3.86717940322408	-5.25699213917495
Cl	2.67359367317736	3.44998710020143	0.50535195911442
Cl	5.59339584564864	2.29519647238645	0.45553686868966
Cl	6.02051730662431	-0.78453777832161	0.16330135816014
Cl	3.52555631634337	-2.68026511714151	-0.08400248726700
O	0.60666380695754	1.35087032048911	0.28137023998861
O	0.95983480637489	-1.19609217886719	0.03167795019436
Sb	-0.83138464327012	-0.06586483045311	0.04281603504134

[3*t*$_C$-F]$^-$ (gas phase)

Sb	0.80088919880218	0.02534939782314	-0.36407054918908
Cl	-3.09544636210791	-3.14685608194220	-0.41645343643051
Cl	-3.18954605930598	3.06732731519958	-0.29851221533332
Cl	-5.84248255597971	-1.63983052273051	-0.60438379799786
Cl	-5.89061605464823	1.48532231628681	-0.54588094294602
F	0.34868262219262	0.07301124390475	-2.26727052318956
O	-0.83576171970517	1.30525127021404	-0.14094200705533
O	-0.78919205846874	-1.32135681280394	-0.21264340483827
C	1.04128884642164	-0.01465047767026	1.79534696066245
C	2.13586676471815	-1.61632349909704	-0.75476638703823
C	-1.95863744074151	-0.73342885574705	-0.28060988846254
C	-1.98243850918229	0.68516988950783	-0.24771806356352
C	2.08665952931948	1.71568094302538	-0.69433467687012
C	3.00468497608438	-2.09397021745060	0.21924781371507
C	3.46500524465657	1.54140123591710	-0.73032812933033
C	3.85681089969514	-3.16045263331130	-0.04368508747460
C	-3.15314841895994	-1.42650399074769	-0.38880963032434
C	3.81347639209127	3.89824104836126	-1.14421775729878
C	4.31579568009304	2.61740956744539	-0.95119485372414
C	2.43239007866006	4.06933318047795	-1.10733097721575
C	0.54806913967662	-1.06731531732714	2.56402106223368
C	-3.19734606454753	1.34552354174958	-0.33147409148707
C	0.73968707355557	-1.09753539998132	3.93744048237218
C	3.85919471039218	-3.78454896769911	-1.28425693822138
C	2.98916785819204	-3.30259421968186	-2.25854617858370
C	2.14329307356607	-2.23582976021671	-2.00277249549417
C	1.57932860938525	2.99841685859090	-0.88888183341310
C	-4.39706173012095	0.63444605617003	-0.43989182305616

C	1.90742730150774	0.97571907091778	3.82742723694633
C	1.71969611312962	1.00617043865432	2.45130568548318
C	-4.37519444976162	-0.74866151760329	-0.46665110437938
C	1.42181495787179	-0.07666143533641	4.59324273055490
C	4.74565132048343	-4.96370238354761	-1.56467916509440
C	4.72473225088542	5.06152920418177	-1.41340068987828
C	1.60110377775897	-0.10082054432786	6.08430966916860
H	3.02275801625165	-1.64312940566072	1.20449219293379
H	3.89587470085795	0.55644574068034	-0.58946638121842
H	4.52515772251144	-3.51255879553109	0.73465979485151
H	5.38772520756809	2.45357461788411	-0.97456782094902
H	2.01275865394653	5.05896719168243	-1.25357831647385
H	0.01090494306775	-1.87903532954759	2.09035538525091
H	0.34950137163026	-1.93266331230375	4.50954512875546
H	2.97135668343754	-3.77122887606119	-3.23678082653691
H	1.48023177214865	-1.88368704757560	-2.77980976009744
H	0.51129005007993	3.16899930338154	-0.86722284407823
H	2.44256359777214	1.78690995528766	4.30948343305539
H	2.11769584531283	1.84396077534779	1.89174327253003
H	4.18125789407688	-5.89827600417340	-1.53269987312080
H	5.55073289285600	-5.04561792708413	-0.83424788630048
H	5.20126440362692	-4.89751029948830	-2.55361035790110
H	4.41188737089664	5.95222321358727	-0.86690143492810
H	4.73201427159429	5.32146921413468	-2.47431105024074
H	5.75309124218226	4.84108769485951	-1.12665211615458
H	0.71024113860622	0.26935682609140	6.59659854456103
H	2.43758575612485	0.52439906673038	6.39777968602326
H	1.78650220984150	-1.11100844344709	6.45138109679186

$[3t_C\text{-F}]^-$ (water phase)

Sb	1.02975458219676	0.17167182983391	-0.82504699580257
Cl	-4.69743533206382	-1.28061268608581	2.61365319665448
Cl	-5.64848902565419	-1.27881240917284	-0.36510149055288
Cl	-3.61747502625961	-0.53208300078446	-2.63563407429936
Cl	-1.73003934904944	-0.51643221896832	3.28608751312468
F	1.84840458941451	0.15070598473875	-2.59384940652181
O	-0.14071465517274	0.16841554419862	0.90950183543768
O	-0.94342381104265	0.16414720304321	-1.61479162630377
C	0.95840974990272	2.31869590141989	-0.90288242363265
C	-1.38701024158658	-0.16790085048456	0.65923590363500
C	2.12140819937222	3.01818056155914	-1.20364940651365
C	-1.81294237515634	-0.17311995901490	-0.69811440565071
C	-0.18435187828290	3.04368795242252	-0.59093402618734
H	-1.11026455540284	2.54051204752705	-0.34762258073104
C	-4.02419136638845	-0.86398623253907	0.03007411864728
C	-2.28419119334645	-0.51916904808458	1.65562573576652
C	-3.12469342756328	-0.52773963802981	-0.98666228551072
C	-0.15931233982945	4.43140339031718	-0.57354241924832
H	-1.06252279744776	4.97454126053917	-0.31754768037949
C	2.84226578199863	0.15405930760945	0.35483770748430
C	-3.60533767608568	-0.86122777525727	1.34947341221976
C	1.00219330681745	5.13515499703001	-0.87160003820875
C	2.14323650853481	4.40525177996670	-1.18797968941397
H	3.06566988291467	4.92679253982081	-1.42001279314950
C	0.89771749494004	-1.97530599995560	-0.90352747354641
C	2.97521553722299	0.97975378531599	1.46631490226040
H	2.17653794068553	1.66029279866957	1.73651445489889
C	1.04684520690153	-2.71206840147132	0.26473055734479
C	5.04564699318788	-0.72583792861324	0.81424700031298
H	5.85413121639307	-1.39725393279946	0.54557425551541
C	3.89192622358110	-0.70108519585943	0.04173033562396
C	0.63523884182262	-2.65325265453961	-2.08775112653908
C	5.17622899433385	0.09244087110195	1.93135505648465
C	4.12325564862703	0.94649658638137	2.24435001985830

H	4.20044742138414	1.59489521263724	3.11056380700885
C	0.53430998243951	-4.03673067621784	-2.10038393495091
H	0.32916979554749	-4.54670808364964	-3.03556464331413
C	0.94530834489400	-4.09613269126318	0.24778127485357
H	1.06364936300839	-4.64987161767306	1.17281870234992
C	0.68488178323192	-4.78020141424159	-0.93382573933266
H	1.24403783933249	-2.21107046116380	1.20512078084137
H	3.03291798661508	2.48296887480185	-1.44137183321217
H	3.81517780541740	-1.36363353477753	-0.81270031028042
H	0.50819252564042	-2.10396036077066	-3.01103973959651
C	1.01790746671559	6.63716134115358	-0.87420990105101
H	1.99240311456454	7.02972768700466	-0.58169699285564
H	0.79426752854838	7.03483956702267	-1.86688871105280
H	0.27636222014818	7.04707685146665	-0.18797271190173
C	6.40189198487096	0.03294236888726	2.79782965892570
H	6.63277297980412	1.00485389820652	3.23507005138631
H	6.26705493859337	-0.66807525444074	3.62493073835600
H	7.27724789629099	-0.29478353287105	2.23661244007174
C	0.53151964550494	-6.27426835337792	-0.94503296836497
H	-0.51443821508850	-6.56363406198897	-0.82049752737596
H	0.87646489187545	-6.70788627367675	-1.88431467069708
H	1.09444690214586	-6.74203843490269	-0.13676489288453

[3t_C-F]$^-$ (chloroform phase)

Sb	1.06936808120358	0.19529462880365	-0.78658277186266
Cl	-4.63958998142840	-1.41151513407555	2.61571340798210
Cl	-5.58878819619652	-1.39909460368749	-0.36541425839874
Cl	-3.57712244947163	-0.56961652230569	-2.62425933499669
Cl	-1.69311894608395	-0.58203275713164	3.29846836172452
F	1.87028459081049	0.19009314920746	-2.57945206694589
O	-0.12343041492708	0.17986411314921	0.93546040980182
O	-0.92490597722110	0.19344318917068	-1.58985102912478
C	0.96898879975741	2.33704201513248	-0.88542933016334
C	-1.36082869461508	-0.18297684289126	0.67761492061188
C	2.11691811737199	3.04828802803184	-1.22188652981797
C	-1.78550613311941	-0.17863426328792	-0.68050164804229
C	-0.17835541229220	3.05170281681974	-0.56043104807406
H	-1.09451986728226	2.54540509569825	-0.28655563484032
C	-3.97832522290120	-0.94517015710181	0.03760687796751
C	-2.24812907656000	-0.57490810052248	1.66771694573461
C	-3.08653152050563	-0.57027523069375	-0.97303959990302
C	-0.17388650494674	4.44051370267980	-0.56840564943586
H	-1.07949690461449	4.97436339579282	-0.30271306769457
C	2.87669859135325	0.16443104952795	0.38539709441832
C	-3.56035232958899	-0.94844939850299	1.35761746105658
C	0.97146615409547	5.15697917836424	-0.90495043046734
C	2.11768374477991	4.43648797231941	-1.23226102196380
H	3.02708093338374	4.96599092458836	-1.49375972268104
C	0.90272631525302	-1.94809245714136	-0.90462943989269
C	3.02918733807571	1.01252336087260	1.48045272733393
H	2.25423023124851	1.72639005050497	1.73534978005093
C	1.01361699101512	-2.70343720214441	0.25852002876522
C	5.04950104832506	-0.77412563401376	0.87927770911304
H	5.83571431380688	-1.47827723247567	0.63158853507162
C	3.90083723971690	-0.72963929558101	0.09684583596178
C	0.64975549049150	-2.60941686171519	-2.10153324808050
C	5.19905332340037	0.06349076729365	1.98016431029891
C	4.16930100169677	0.95873988639000	2.26771252567040
H	4.25962155379120	1.62085081314703	3.12196823528481
C	0.51907926537971	-3.99172897279511	-2.13220329429037
H	0.32666611542112	-4.48557878777732	-3.07824855924406
C	0.88099820382140	-4.08524344708560	0.22459032113213
H	0.97301332594452	-4.65065088943539	1.14528892924562
C	0.62959157131803	-4.75295664590694	-0.97097119918291

H	1.20862061516537	-2.22400817912637	1.21095194890791
H	3.03508209094162	2.52784484007678	-1.46887072267996
H	3.81445285685693	-1.41187950249947	-0.74145059866915
H	0.55442511294484	-2.05713856560135	-3.02744766752035
C	0.96438786406900	6.65690209684911	-0.93601512013550
H	1.93648232630098	7.06992917954327	-0.66557176440117
H	0.72403101290731	7.02884213680481	-1.93475656738504
H	0.22251125524767	7.06916770631997	-0.25186465128123
C	6.42668463800559	0.01403526640843	2.84110959259236
H	6.97533153749831	0.95729126518500	2.80681027338626
H	6.17138335371509	-0.16704735134811	3.88675673990799
H	7.10830929080637	-0.77520453826781	2.52517388248621
C	0.45237266619682	-6.24253218816643	-1.00115166932026
H	-0.58854776281439	-6.51800881331289	-0.81618193382568
H	0.72859983502564	-6.66170030499061	-1.96880278741859
H	1.05506444742570	-6.73477928909607	-0.23744554676664

[3t_O-F]$^-$ (gas phase)

Sb	1.02975458219676	0.17167182983391	-0.82504699580257
Cl	-4.69743533206382	-1.28061268608581	2.61365319665448
Cl	-5.64848902565419	-1.27881240917284	-0.36510149055288
Cl	-3.61747502625961	-0.53208300078446	-2.63563407429936
Cl	-1.73003934904944	-0.51643221896832	3.28608751312468
F	1.84840458941451	0.15070598473875	-2.59384940652181
O	-0.14071465517274	0.16841554419862	0.90950183543768
O	-0.94342381104265	0.16414720304321	-1.61479162630377
C	0.95840974990272	2.31869590141989	-0.90288242363265
C	-1.38701024158658	-0.16790085048456	0.65923590363500
C	2.12140819937222	3.01818056155914	-1.20364940651365
C	-1.81294237515634	-0.17311995901490	-0.69811440565071
C	-0.18435187828290	3.04368795242252	-0.59093402618734
H	-1.11026455540284	2.54051204752705	-0.34762258073104
C	-4.02419136638845	-0.86398623253907	0.03007411864728
C	-2.28419119334645	-0.51916904808458	1.65562573576652
C	-3.12469342756328	-0.52773963802981	-0.98666228551072
C	-0.15931233982945	4.43140339031718	-0.57354241924832
H	-1.06252279744776	4.97454126053917	-0.31754768037949
C	2.84226578199863	0.15405930760945	0.35483770748430
C	-3.60533767608568	-0.86122777525727	1.34947341221976
C	1.00219330681745	5.13515499703001	-0.87160003820875
C	2.14323650853481	4.40525177996670	-1.18797968941397
H	3.06566988291467	4.92679253982081	-1.42001279314950
C	0.89771749494004	-1.97530599995560	-0.90352747354641
C	2.97521553722299	0.97975378531599	1.46631490226040
H	2.17653794068553	1.66029279866957	1.73651445489889
C	1.04684520690153	-2.71206840147132	0.26473055734479
C	5.04564699318788	-0.72583792861324	0.81424700031298
H	5.85413121639307	-1.39725393279946	0.54557425551541
C	3.89192622358110	-0.70108519585943	0.04173033562396
C	0.63523884182262	-2.65325265453961	-2.08775112653908
C	5.17622899433385	0.09244087110195	1.93135505648465
C	4.12325564862703	0.94649658638137	2.24435001985830
H	4.20044742138414	1.59489521263724	3.11056380700885
C	0.53430998243951	-4.03673067621784	-2.10038393495091
H	0.32916979554749	-4.54670808364964	-3.03556464331413
C	0.94530834489400	-4.09613269126318	0.24778127485357
H	1.06364936300839	-4.64987161767306	1.17281870234992
C	0.68488178323192	-4.78020141424159	-0.93382573933266
H	1.24403783933249	-2.21107046116380	1.20512078084137
H	3.03291798661508	2.48296887480185	-1.44137183321217
H	3.81517780541740	-1.36363353477753	-0.81270031028042
H	0.50819252564042	-2.10396036077066	-3.01103973959651
C	1.01790746671559	6.63716134115358	-0.87420990105101
H	1.99240311456454	7.02972768700466	-0.58169699285564

H	0.79426752854838	7.03483956702267	-1.86688871105280
H	0.27636222014818	7.04707685146665	-0.18797271190173
C	6.40189198487096	0.03294236888726	2.79782965892570
H	6.63277297980412	1.00485389820652	3.23507005138631
H	6.26705493859337	-0.66807525444074	3.62493073835600
H	7.27724789629099	-0.29478353287105	2.23661244007174
C	0.53151964550494	-6.27426835337792	-0.94503296836497
H	-0.51443821508850	-6.56363406198897	-0.82049752737596
H	0.87646489187545	-6.70788627367675	-1.88431467069708
H	1.09444690214586	-6.74203843490269	-0.13676489288453
